# Molecular evolutionary engineering of xylose isomerase to improve its catalytic activity and performance of micro-aerobic glucose/xylose co-fermentation in *Saccharomyces cerevisiae*

**DOI:** 10.1186/s13068-019-1474-z

**Published:** 2019-06-06

**Authors:** Taisuke Seike, Yosuke Kobayashi, Takehiko Sahara, Satoru Ohgiya, Yoichi Kamagata, Kazuhiro E. Fujimori

**Affiliations:** 1 Bioproduction Research Institute (BPRI), National Institute of Advanced Industrial Science and Technology (AIST), 1-1-1 Higashi, Tsukuba, Ibaraki 305-8566 Japan; 2 Bioproduction Research Institute (BPRI), National Institute of Advanced Industrial Science and Technology (AIST), 2-17-2-1 Tsukisamu-higashi, Toyohira, Sapporo, Hokkaido 062-8517 Japan; 3 Present Address: Center for Biosystems Dynamics Research (BDR), RIKEN, 6-2-3 Furuedai, Suita, Osaka 565-0874 Japan; 4 Present Address: Biomaterial in Tokyo Company Limited, 4-7 Kashiwa-Inter-Minami, Kashiwa, Chiba 277-0872 Japan

**Keywords:** *Saccharomyces cerevisiae*, Xylose isomerase, *Lachnoclostridium phytofermentans*, Mutagenesis, Bioethanol, Error-prone PCR, Metabolic engineering

## Abstract

**Background:**

Expression of d-xylose isomerase having high catalytic activity in *Saccharomyces cerevisiae* (*S. cerevisiae*) is a prerequisite for efficient and economical production of bioethanol from cellulosic biomass. Although previous studies demonstrated functional expression of several xylose isomerases (XI) in *S. cerevisiae*, identification of XIs having higher catalytic activity is needed. Here, we report a new strategy to improve xylose fermentation in the *S. cerevisiae* strain IR-2 that involves an evolutionary engineering to select top-performing XIs from eight previously reported XIs derived from various species.

**Results:**

Eight XI genes shown to have good expression in *S. cerevisiae* were introduced into the strain IR-2 having a deletion of *GRE3* and *XKS1* overexpression that allows use of d-xylose as a carbon source. Each transformant was evaluated under aerobic and micro-aerobic culture conditions. The strain expressing XI from *Lachnoclostridium phytofermentans* ISDg (*Lp*XI) had the highest d-xylose consumption rate after 72 h of micro-aerobic fermentation on d-glucose and d-xylose mixed medium. To enhance *Lp*XI catalytic activity, we performed random mutagenesis using error-prone polymerase chain reaction (PCR), which yielded two *Lp*XI candidates, SS82 and SS92, that showed markedly improved fermentation performance. The *Lp*XI genes in these clones carried either T63I or V162A/N303T point mutations. The SS120 strain expressing *Lp*XI with the double mutation of T63I/V162A assimilated nearly 85 g/L d-glucose and 35 g/L d-xylose to produce 53.3 g/L ethanol in 72 h with an ethanol yield of approximately 0.44 (g/g-input sugars). An in vitro enzyme assay showed that, compared to wild-type, the *Lp*XI double mutant in SS120 had a considerably higher *V*_max_ (0.107 µmol/mg protein/min) and lower *K*_m_ (37.1 mM).

**Conclusions:**

This study demonstrated that *Lp*XI has the highest d-xylose consumption rate among the XIs expressed in IR-2 under micro-aerobic co-fermentation conditions. A combination of novel mutations (T63I and V162A) significantly improved the enzymatic activity of *Lp*XI, indicating that *Lp*XI-*T63I/V162A* would be a potential construct for highly efficient production of cellulosic ethanol.

**Electronic supplementary material:**

The online version of this article (10.1186/s13068-019-1474-z) contains supplementary material, which is available to authorized users.

## Background

Industrial production of second-generation bioethanol (also called cellulosic ethanol) produced from lignocellulosic biomass such as corn stover, rice straw or sugarcane bagasse is critical to avoid competition with food crops that are predominantly used for production of first-generation bioethanol [1]. Despite various trials, commercial production of cellulosic ethanol still faces technological difficulties in sustainable supply of raw materials, delignification to liberate cellulose and hemicellulose from these complexes, effective carbohydrate depolymerization to produce free sugars and fermentation of mixed sugars to produce ethanol as well as high production costs [2]. In particular, the insufficient ability of microorganisms to metabolize pentose sugars, particularly d-xylose, which comprises a substantial portion of herbaceous hemicelluloses, is associated with low yields of bioethanol from lignocellulosic biomass [2, 3]. The budding yeast *Saccharomyces cerevisiae* (*S. cerevisiae*) is a potential host candidate for industrial ethanol production due to its high fermentation activity, resistance to ethanol and robustness in the presence of compounds that inhibit fermentation. Significant efforts have been made over the past two decades to develop engineered *S. cerevisiae* strains having modified pathways that enhance d-xylose metabolism, but the critical genes needed to optimize d-xylose metabolism in yeast remain unclear.

Two different metabolic pathways have been proposed for the initial conversion step of d-xylose by *S. cerevisiae* [4]. The first, a redox pathway catalyzed by NADPH-dependent xylose reductase (XR) followed by NAD^+^-dependent xylitol dehydrogenase (XDH), involves different coenzyme specificities of XR and XDH that cause a co-factor imbalance and subsequent accumulation of byproduct xylitol. Although attempts to address this problem including adaptive evolution, alteration of co-factor dependency and fine-tuning of enzyme expression levels have been partially successful in reducing xylitol production [5–9], the accumulation of xylitol remains problematic. The second pathway is the direct isomerization of d-xylose by d-xylose isomerase (XI), which would be superior to the redox pathway, since co-factor imbalance and xylitol accumulation do not occur. However, XI-based pathways predominate in bacteria and these enzymes are difficult to express functionally in yeast. The first attempts to obtain bacterial XIs encoded by *xylA* genes that can function in *S. cerevisiae* were unsuccessful, likely due to improper folding and cytoplasmic insolubility of the expressed protein [10–12]. In 1996, Walfridsson et al. [13] first reported that XI from the extreme thermophiles *Thermus thermophilus* could be expressed in an active form in *S. cerevisiae*, although the optimal temperature of the XI for function, 85 °C, was unsuitable for practical applications and only 4% of maximum activity was retained at 30 °C. Later, the first XI from the anaerobic fungus *Piromyces* sp. E2 was expressed in yeast, but the recombinant strain consumed d-xylose slowly [14]. Successful expression of XIs in *S. cerevisiae* was subsequently reported by several research groups in succession: *Orpinomyces* sp. ukk1 [15–17], *Lachnoclostridium* (previously known as *Clostridium*) *phytofermentans* ISDg [18, 19], *Ruminococcus flavefaciens* 17 [20], *Prevotella ruminicola* TC2-24 [21], *Burkholderia cenocepacia* J2315 [22, 23], *Ruminiclostridium* (previously known as *Clostridium*) *cellulolyticum* H10 [24] and *Streptomyces rubiginosus* [25]. Although the recombinant *S. cerevisiae* strains expressing the different XIs functioned to some extent, which XIs would be best suited for industrial ethanol production was still unclear.

In 2012, Lee and colleagues [26] subjected XI from *Piromyces* sp. E2 to three rounds of directed evolution and generated XI mutants containing six mutations (E15D, E114G, E129D, T142S, A177T and V433I) that had increased d-xylose consumption rates and in turn improved aerobic growth rates and ethanol production. The mutated XI exhibited a 77% increase in the *V*_max_ value and a twofold lower *K*_m_ compared to the wild-type. Back-substitution analyses showed that the E15D and T142S mutations in particular contributed to the improved catalytic activity of this XI [26]. Site-directed mutagenesis was also used to improve the XI enzyme activity of *R. flavefaciens* [20]. A G179A mutation, at a position close to the d-xylose binding site, showed a 15% increase in activity over the corresponding wild-type, and the 5′-P10 modification, in which the first 10 amino acids are replaced by the corresponding 12 amino acids from *Piromyces* sp. E2 XI, produced a 26.8% increase in activity over the wild-type while maintaining a *K*_m_ value of 66.7 mM [20]. In addition, Waltman and colleagues [25] carried out rational enzyme engineering of *S. rubiginosus* XI to produce several variants (e.g., D215N) that show significantly lower affinity for d-xylose at < pH 6. Although these mutated XIs have improved performance in anaerobic fermentation, they should be reexamined in a common industrial strain under identical fermentation conditions. In this study, we evaluated the catalytic activities of previously reported XIs under identical fermentation conditions using a common parental strain SS29, a haploid strain derived from the diploid strain IR-2 that has a deletion of the endogenous xylose reductase *GRE3*, and directed evolution of genes encoding *Lp*XI to improve the catalytic activities of XI for efficient and cost-effective bioethanol production from cellulosic biomass.

## Results

### Growth kinetics of transformants expressing XIs in d-xylose medium under aerobic conditions

A list of strains used in this study is shown in Additional file 1: Table S1. Eight currently known XI genes (*xylA*) derived from microorganisms were expressed in yeast and the ability of the transformants to metabolize d-xylose was assessed. Codon-optimization of XI gene nucleotide sequences was performed to maximize translational efficiency in *S. cerevisiae* and the genes were cloned into the low copy number expression vector pUG35. The XI genes under the control of the stress-inducible *HSP12* promoter and an additional xylulokinase gene (*XKS1*) under the control of the strong promoter *PGK1* were expressed in the *S. cerevisiae* strain SS29 with disrupted endogenous xylose reductase gene (*GRE3*). Although the function of *GRE3* in consumption of d-xylose by *S. cerevisiae* is unclear, we nonetheless disrupted this gene to ensure that it would not compete with the exogenous XI during d-xylose metabolism. In addition, to maintain the enhanced d-xylose metabolic flow by the introduced XIs, we increased the expression level of *XKS1* using a strong *PGK1* promoter. These plasmids carrying the eight different XIs and a control vector lacking XI genes were used to transform the host strain SS29 derived from the diploid IR-2 to generate the strains termed SS36 to SS44 (see “Methods” section).

**Fig. 1:**
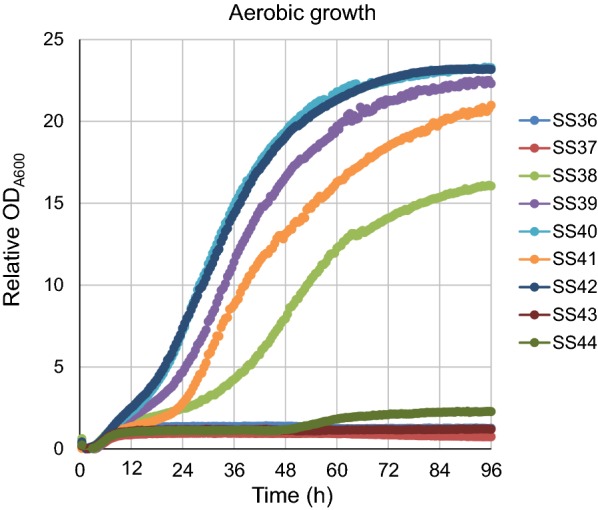
Growth kinetics of a control strain without XI and strains containing plasmid vector expressing various XIs in xylose media as the sole carbon source. Strains were cultured under aerobic conditions using YPX_50_ medium containing G418 (200 µg/mL). Cells for strain SS36 (blue), SS37 (red), SS38 (yellow green), SS39 (purple), SS40 (light blue), SS41 (orange), SS42 (dark blue), SS43 (brown) and SS44 (green) were initially inoculated at 0.1 U OD_A600_ and grown at 30 °C with shaking at 1000 rpm. Growth was measured using an Infinite 200 PRO microplate reader. Cell density was measured hourly. The dots indicate mean value of biological triplicates

We first examined the growth kinetics of each strain in high-concentration d-xylose media YPX_50_ under aerobic conditions (Fig. 1). Among the XI-expressing strains, SS40 (*Osp*XI) had the highest specific growth rate of 0.71 h^−1^ (Table 1). SS42 (*Lp*XI) exhibited similar, but slower, growth kinetics to SS40 with a specific growth rate of 0.62 h^−1^ (Fig. 1 and Table 1). SS38 (*Pr*XI), SS39 (*Rf*XI) and SS41 (*Psp*XI) showed aerobic growth on xylose media but had lower growth rates than did SS40 and SS42 (Table 1). SS37 (*Bc*XI), SS43 (*Rc*XI), SS44 (*Sr*XI) and the control strain SS36 grew poorly or at undetectable levels under these culture conditions (Table 1).

**Table 1:** Comparison of specific aerobic growth rates in YPX_50_ medium for strains harboring XIs

Strain	Name	Origin	Specific growth rates (µ) (h^−1^)
SS36	^a^	^a^	NA
SS37	*Bc*XI	*Burkholderia cenocepacia* J2315	NA
SS38	*Pr*XI	*Prevotella aff. ruminicola* TC2-24	0.39 ± 0.05
SS39	*Rf*XI	*Ruminococcus flavefaciens* 17	0.58 ± 0.03
SS40	*Osp*XI	*Orpinomyces* sp. ukk1	0.71 ± 0.07
SS41	*Psp*XI	*Piromyces* sp. E2	0.51 ± 0.10
SS42	*Lp*XI	*Lachnoclostridium phytofermentans* ISDg	0.62 ± 0.09
SS43	*Rc*XI	*Ruminiclostridium cellulolyticum* H10	NA
SS44	*Sr*XI	*Streptomyces rubiginosus*	0.03 ± 0.01

The mean specific growth rate with standard deviations of biological triplicates are shown

NA: aerobic growth in YPX_50_ medium was very poor or showed no growth

^a^SS36 was a control strain carrying a plasmid vector lacking any XI genes

### Comparison of xylose consumption and ethanol production of wild-type XI-expressing strains in glucose and xylose co-fermentation under micro-aerobic conditions

**Fig. 2:**
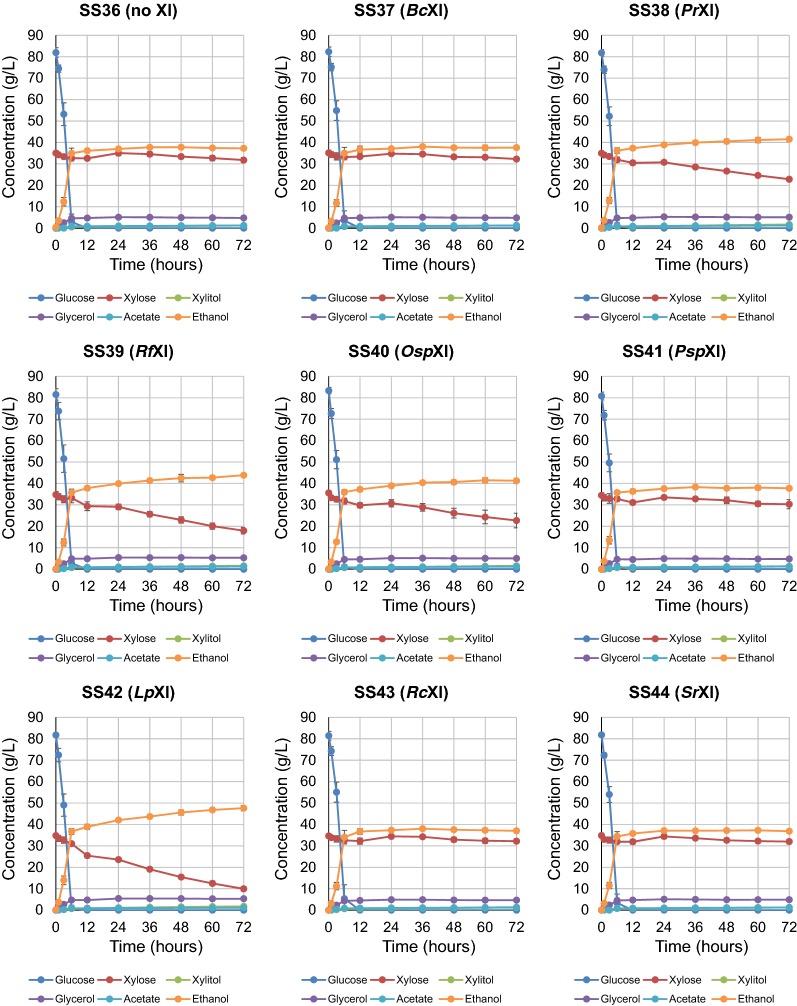
Fermentation performance of a control strain without XI and strains containing plasmid vectors expressing various XIs in glucose/xylose co-fermentation under micro-aerobic conditions. Batch fermentation examinations were performed using cultures inoculated with cells at high density (OD_A600_ = 20) under micro-aerobic conditions. A control strain lacking XI (SS36) and eight strains expressing XIs (SS37 to SS44) were grown in 70 mL YPD_85_X_35_ medium containing G418 (200 µg/mL) at 30 °C. Samples of culture medium were collected at 0, 1, 3, 6, 12, 24, 36, 48, 60 and 72 h (10 times) for HPLC analysis of metabolites. The XIs were introduced episomally via low copy number plasmids into the same parental strain, SS29. The dots and error bars in the panels represent the mean concentrations and standard deviations, respectively, of the following metabolites measured in biological triplicates: glucose (blue), xylose (red), xylitol (yellow green), glycerol (purple), acetate (light blue) and ethanol (orange). The details for the metabolite concentrations are shown in Additional file 2: Table S2

The fermentation activity of each XI-expressing strain was evaluated in the d-glucose and d-xylose mixed medium YPD_85_X_35_ at high cell density (initial OD_A600_ = 20 for each) and 30 °C under micro-aerobic conditions. During the fermentation, changes in residual sugar concentrations (d-glucose and d-xylose) and metabolic products (xylitol, glycerol, acetate and ethanol) were measured by high-performance liquid chromatography (HPLC). Although all tested strains consumed d-glucose within 12 h, differences were observed in d-xylose consumption and ethanol production after glucose depletion. Among the XI-expressing strains, SS42 (*Lp*XI) showed the most efficient d-xylose consumption and ethanol production with 47.6 g/L ethanol produced from 85 g/L d-glucose and 35 g/L d-xylose in 72 h. The amount of residual d-xylose was dramatically decreased to 10 g/L in 72 h relative to the control strain SS36 (Fig. 2 and Additional file 2: Table S2). SS39 (*Rf*XI) produced 43.9 g/L ethanol with consumption of approximately 17 g/L d-xylose (Fig. 2 and Additional file 2: Table S2). Unexpectedly, SS40 (*Osp*XI), which showed the fastest growth under aerobic conditions in YPX_50_ medium, had poor fermentation performance under these conditions. SS40 consumed only 12.6 g/L d-xylose in 72 h, approximately half that consumed by SS42 (Fig. 2 and Additional file 2: Table S2). SS38 (*Pr*XI) behaved similarly to SS40 (*Osp*XI) (Fig. 2 and Additional file 2: Table S2). The other four strains SS37 (*Bc*XI), SS41 (*Psp*XI), SS43 (*Rc*XI) and SS44 (*Sr*XI) consumed essentially the same amount of d-xylose as the control strain SS36 and no improvement in xylose consumption and ethanol production was observed with XI expression (Fig. 2 and Additional file 2: Table S2).

### Comparison of xylose consumption and ethanol productivity by strains expressing previously characterized XI mutations in glucose and xylose co-fermentation under micro-aerobic conditions

Several amino acids substitutions identified in directed evolution studies of *Rf*XI and *Psp*XI were recently found to improve xylose metabolism [20, 25]. We thus compared the fermentation performance of strains expressing *Rf*XI and *Psp*XI carrying these known mutations under the same conditions. The resulting strains, SS45 (*Rf*XI-*G179A*), SS46 (*Psp*XI-*E15D*) and SS49 (*Psp*XI-*N370S*), exhibited better fermentation performance than the parental strains SS39 (*Rf*XI) and SS41 (*Psp*XI), respectively, in co-fermentation with d-glucose and d-xylose (Fig. 2 and Additional file 2: Tables S2, Additional file 3: Figure S1 and Additional file 4: Table S3). However, these mutations had little effect on d-xylose consumption. Moreover, no improvement in d-xylose consumption by strains SS47 (*Psp*XI-*C54R*), SS48 (*Psp*XI-*T142S*), SS50 (*Psp*XI-*C54R/N370S*) and SS51 (*Psp*XI-*E51D/T142S*) was observed (Additional file 3: Figure S1 and Additional file 4: Table S3). The ethanol production and d-xylose consumption of SS42 (*Lp*XI) were substantially better than those of SS45 (*Rf*XI-*G179A*), SS46 (*Psp*XI-*E15D*) and SS49 (*Psp*XI-*N370S*).

### Identification of beneficial *Lp*XI mutations and characterization of growth and fermentation properties of resulting clones

Based on the above results for the eight XI genes, *Lp*XI was chosen for expression in *S. cerevisiae* and used for protein engineering to improve its catalytic function. We carried out random mutagenesis of the *Lp*XI gene using error-prone PCR. In brief, plasmids containing mutated *Lp*XI were introduced into the parental strain SS29 and the growth activity on plates containing higher concentrations of d-xylose (80 g/L d-xylose, YPX_80_) was examined. Approximately, 2.4 × 10^5^ yeast transformants were obtained, and in a growth assay on YPX_80_ plates, 24 colonies grew well. Repeated examination of growth assay on YPX_80_ plates to eliminate false-positive clones from the *Lp*XI mutant library yielded 11 transformants as evolved strains containing mutated *Lp*XI. These 11 *Lp*XI expression plasmids were prepared from the transformants and the nucleotide sequences of the *Lp*XI coding regions were determined by Sanger sequencing (Table 2). The aerobic growth properties and fermentation activity in micro-aerobic co-fermentation of d-glucose and d-xylose for the transformants were evaluated under the same experimental condition as described above. In the growth assay, all candidates showed improved growth phenotypes even on high-concentration xylose plates and liquid media containing 80 g/L xylose as the sole carbon source compared to that for the control strain SS42 (Table 2). In particular, the mutant clone M6-13 (*Lp*XI-*N233I*, episomally) had approximately 4.0-fold higher specific growth rates relative to the parental strain SS42 (Table 2). A fermentation assay indicated that all 11 clones had superior fermentation ability, as evidenced by exhaustion of the input d-xylose within 72 h and production of at least 52 g/L ethanol relative to the control strain SS42 (wild-type *Lp*XI, episomally) (Fig. 2 and Additional file 2: Table S2, Additional file 5: Figure S2 and Additional file 6: Table S4).

**Table 2:** Mutations identified in *Lp*XI-expressing plasmids and relative specific growth rate (µ/µ_0_) of isolated clones in YPX_80_ medium under aerobic cultivations

Clone	Substitutions in *Lp*XI	Specific growth rate (µ) (h^−1^)^a^	Relative growth rate (µ/µ_0_)
M6-13	*N223I*	1.07 ± 0.06	3.97
M6-11	*D207G*	0.98 ± 0.12	3.63
M6-21	*R191K/E192K*	0.95 ± 0.11	3.54
M6-22	*L304S*	0.93 ± 0.09	3.44
M6-15	*L78S*	0.92 ± 0.18	3.43
M6-7	*Y13H/D228* *V/P233P*	0.86 ± 0.16	3.20
M6-10	*T273A*	0.82 ± 0.07	3.06
M6-6	*T133T/K136T/A176T*	0.82 ± 0.31	3.04
M6-19	*E114G*	0.75 ± 0.08	2.80
M6-20	*V162A/T273T/N303T*	0.49 ± 0.03	1.81
M6-2	*T63I/A121A*	0.30 ± 0.03	1.13
SS42	Wild-type	0.27 ± 0.00	1.00

^a^Mean ± standard deviations

### Micro-aerobic co-fermentation of d-glucose and d-xylose by strains carrying wild-type and mutated *Lp*XI at the *AUR1* locus

**Fig. 3:**
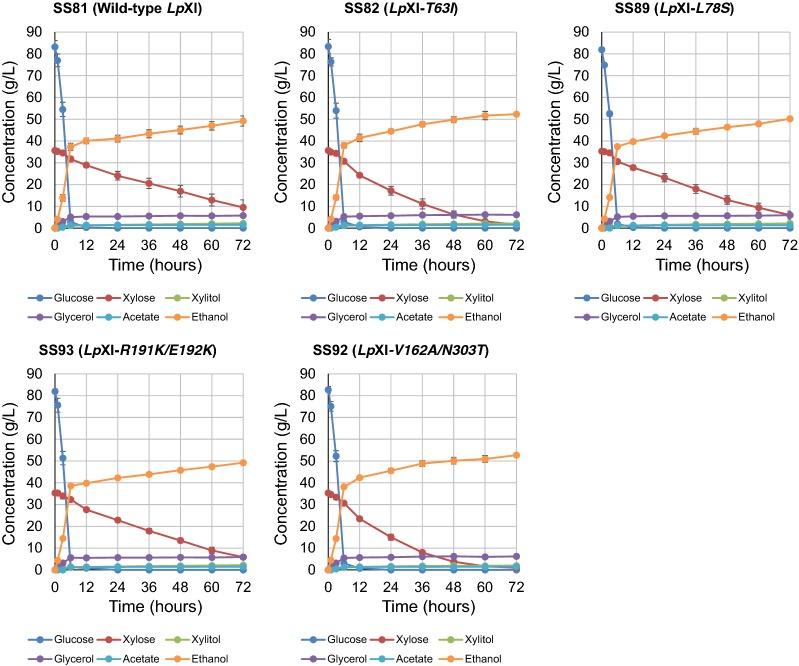
Fermentation performance of wild-type strains and strains with mutated *Lp*XIs integrated into the chromosome in glucose/xylose co-fermentation under micro-aerobic conditions. Batch fermentation examinations were performed under the same conditions as in Fig. 2. The fermentation properties of control strain SS81 (wild-type *Lp*XI) and four representative strains having improved d-xylose consumption rates are shown: SS82 (*Lp*XI-*T63I/A121A*), SS89 (*Lp*XI-*L78S*), SS92 (*Lp*XI-*V162A/T273T/N303T*) and SS93 (*Lp*XI-*R191K/E192K*). The mutated *Lp*XI expression cassettes were introduced into the *AUR1* locus of the chromosome of the parental strain SS29 via homologous recombination. The dots and error bars in the panels represent the mean concentrations and standard deviations, respectively, of the following metabolites in biological triplicates: glucose (blue), xylose (red), xylitol (yellow green), glycerol (purple), acetate (light blue) and ethanol (orange). The details for the metabolite concentrations are shown in Additional file 7: Table S5

As shown above, strains that grow well under aerobic culture conditions and have effective performance in micro-aerobic co-fermentation of d-glucose and d-xylose were successfully obtained, and the amino acid substitutions in the mutated XI were identified. To verify critical mutations for improved fermentation activity in micro-aerobic co-fermentation of d-glucose and d-xylose, wild-type and mutated *Lp*XIs with *XKS1* were integrated at the *AUR1* chromosome locus in SS29. The fermentation performance of the control strain SS81 (wild-type *Lp*XI) was roughly the same as that of SS42 (wild-type *Lp*XI, episomally) with 12.2 g/L and 47.7 g/L of d-xylose and ethanol, respectively, produced in 72 h (Figs. 2 and 3 and Additional file 2: Table S2 and Additional file 7: Table S5). Of the 11 transformants, SS82 (*Lp*XI-*T63I/A121A*, corresponding to the M6-2 clone) and SS92 (*Lp*XI-*V162A/T273T/N303T*, corresponding to the M6-20 clone) showed remarkable improvement in xylose consumption and ethanol productivity in micro-aerobic co-fermentation of d-glucose and d-xylose compared to the control strain SS81 (wild-type *Lp*XI). Within 72 h, SS82 and SS92 both assimilated almost all available d-xylose, with residual d-xylose dramatically reduced to 2.51 g/L and 1.45 g/L, concurrent with the production of 52.2 g/L and 52.4 g/L ethanol, respectively (Fig. 3 and Additional file 7: Table S5). The other strains SS89 (*Lp*XI-*L78S*, corresponding to the M6-15 clone) and SS93 (*Lp*XI-*R191K/E192K*, corresponding to the M6-21 clone) also showed a slight improvement in d-xylose consumption and ethanol production (Fig. 3 and Additional file 7: Table S5). The other 7 strains, SS84 (*Lp*XI-*T133T/K136T/A176T*, corresponding to the M6-6 clone), SS85 (*Lp*XI-*Y13H*/*D228V/P233P*, corresponding to the M6-7 clone), SS86 (*Lp*XI-*T273A*, corresponding to the M6-10 clone), SS87 (*Lp*XI-*D207G*, corresponding to the M6-11 clone), SS88 (*Lp*XI-*N223I*, corresponding to the M6-13 clone), SS91 (*Lp*XI-*E114G*, corresponding to the M6-19 clone) and SS94 (*Lp*XI-*L304S*, corresponding to the M6-22 clone), showed no significant improvement in d-xylose consumption even though the growth of the corresponding clones carrying the mutated *Lp*XI plasmids was much faster than that of the control strain in high-concentration xylose media under aerobic conditions and the performance of glucose/xylose co-fermentation was high (Additional file 8: Figure S3 and Additional file 9: Table S6).

### Characterization of *Lp*XI mutation(s) in SS82 (*Lp*XI-*T63I/A121A*) and SS92 (*Lp*XI-*V162A*/*T273T/N303T*)

**Fig. 4:**
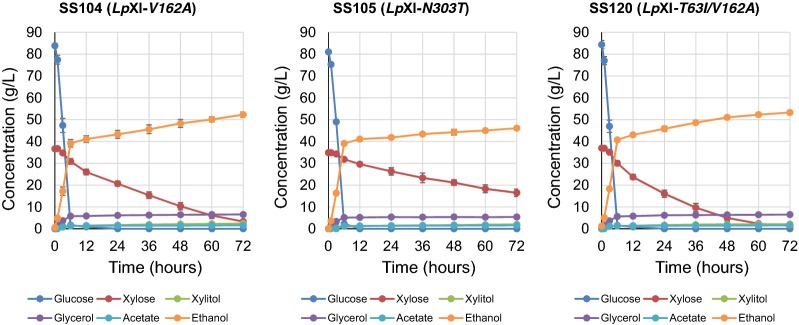
Fermentation performance of strains with single and double mutations in *Lp*XI integrated into the chromosome in glucose/xylose co-fermentation under micro-aerobic conditions. Batch fermentation examinations were performed under the same conditions as for Figs. 2 and 3. The fermentation properties of strains are shown for SS104 (*Lp*XI-*V162A*, single mutation, from SS92), SS105 (*Lp*XI-*N303T*, single mutation, from SS92) and SS120 (*Lp*XI-*T63I/V162A*, combination of mutations from SS82 and SS92, respectively). The mutated *Lp*XI expression cassettes were introduced into the *AUR1* locus of the chromosome of the parental strain SS29 via homologous recombination. The dots and error bars in the panels represent the mean concentrations and standard deviations, respectively, of the following metabolites in biological triplicates: glucose (blue), xylose (red), xylitol (yellow green), glycerol (purple), acetate (light blue) and ethanol (orange). The details for the metabolite concentrations are shown in Additional file 10: Table S7

Both SS82 (*Lp*XI-*T63I/A121A*) and SS92 (*Lp*XI-*V162A*/*T273T/N303T*) showed higher fermentation activity in micro-aerobic co-fermentation of d-glucose and d-xylose, but it was unclear which mutation(s) was responsible for the improvement in d-xylose catalytic activity. The nucleotide variations at residues 121 and 273 in SS82 and SS92 produced no amino acid substitution. As such, we focused on three amino acid substitutions T63I, V162A and N303T, and determined whether one, two or all three mutations would be required to provide beneficial effects for d-xylose consumption and ethanol production in micro-aerobic co-fermentation of d-glucose and d-xylose. The fermentation performance of SS104 (*Lp*XI-*V162A*) was obviously higher than that of the control strain SS81 (wild-type *Lp*XI) but did not exceed that of SS92 (*Lp*XI-*V162A*/*T273T/N303T*) (Figs. 3 and 4 and Additional file 7: Table S5 and Additional file 10: Table S7). On the other hand, strain SS105 (*Lp*XI-*N303T*) showed no improvement in fermentation performance, and the amount of d-xylose consumption was much less than that of the control strain SS81 (wild-type *Lp*XI) (Figs. 3 and 4 and Additional file 7: Table S5 and Additional file 10: Table S7). Furthermore, SS120 (*Lp*XI-*T63I/V162A*) showed superb fermentation ability as evidenced by production of 53.3 g/L ethanol and consumption of nearly all available d-xylose within 72 h. SS92 (*Lp*XI-*V162A*/*T273T/N303T*) and SS120 (*Lp*XI-*T63I/V162A*) had the fastest rates of d-xylose consumption among all the strains tested (Fig. 4). A comparison of fermentation indices showed a d-xylose consumption rate in 36 h (C_xyl36_) for SS82, SS92 and SS120 of 0.68 g/L/h, 0.76 g/L/h and 0.75 g/L/h and 0.47 g/L/h, 0.48 g/L/h and 0.49 g/L/h, respectively, at 72 h (C_xyl72_) (Table 3), which were calculated as increases of 57%, 58% and 64%, respectively, over that of control strain SS81 (C_xyl72_, 0.30 g/L/h). The ethanol yield of these three strains reached 0.44 g/g-input sugars in 72 h (Table 3). The strain carrying the single mutation, SS105 (*Lp*XI-*N303T*), had no improvement in any of the indices. The responsible mutation in SS92 was thus V162A, as N303T alone did not improve fermentation efficiency. Taken together, we concluded that *Lp*XI carrying the double mutation T63I/V162A is the most suitable mutant for functional expression in yeast and for optimal glucose/xylose co-fermentation.

**Table 3:** Comparison of fermentation performance of improved strains with mutated *Lp*XI

Strain	*Lp*XI	C_xyl36_ (g/L/h)^a^	C_xyl72_ (g/L/h)^b^	Y_eth72_ (g/g-input sugars)^c^
SS81	Wild-type	0.36 ± 0.08	0.30 ± 0.08	0.39 ± 0.03
SS82	*T63I/A121A*	0.68 ± 0.06	0.47 ± 0.01	0.44 ± 0.01
SS92	*V162A/T273T/N303T*	0.76 ± 0.04	0.48 ± 0.02	0.44 ± 0.01
SS104	*V162A*	0.59 ± 0.05	0.46 ± 0.01	0.43 ± 0.01
SS105	*N303T*	0.32 ± 0.06	0.25 ± 0.02	0.40 ± 0.01
SS120	*T63I/V162A*	0.75 ± 0.05	0.49 ± 0.00	0.44 ± 0.01

^a^C_xyl36_: d-xylose consumption rate in 36 h

^b^C_xyl72_: d-xylose consumption rate in 72 h

^c^Y_eth72_: ethanol yield in 72 h per input sugars

### Determination of catalytic activities of mutated *Lp*XIs

To confirm the fermentation assay results and determine the underlying mechanisms associated with improved *Lp*XI performance, the catalytic activities of wild-type and mutated *Lp*XIs were assessed using an in vitro enzymatic assay. In brief, total proteins were extracted from each strain expressing wild-type and mutated *Lp*XIs after 24 h of micro-aerobic co-fermentation and the total protein concentration was adjusted to 1 mg/mL before XI activity was measured in terms of decreases in the amount of NADH (see “Methods”). *Lp*XI-*T63I/A121A* (SS82) had a 44% increase in the catalytic activity (*V*_max_) over wild-type and a *K*_m_ value of 29.3 mM. On the other hand, the *V*_max_ for *Lp*XI-*V162A/T273T/N303T* (SS92) showed no increase in catalytic activity over wild-type, but the *K*_m_ of 28.4 mM was significantly lower than the 41.8 mM for wild-type (Table 4, Additional file 11: Table S8). Notably, *Lp*XI-*T63I/V162A* (SS120) showed the highest *V*_max_ among the mutated *Lp*XIs (Table 4).

**Table 4:** Kinetic properties of wild-type and mutated *Lp*XIs

Strain	*Lp*XI	*V*_max_^a^	*K*_m_ (mM)
SS81	Wild-type	0.064 ± 0.002	41.8 ± 0.8
SS82	*T63I/A121A*	0.092 ± 0.001	29.3 ± 2.6
SS92	*V162A/T273T/N303T*	0.066 ± 0.001	28.4 ± 2.3
SS104	*V162A*	0.063 ± 0.002	29.5 ± 2.2
SS105	*N303T*	0.030 ± 0.001	19.1 ± 0.6
SS120	*T63I/V162A*	0.107 ± 0.002	37.1 ± 2.3

The mean and standard deviations of biological triplicates are indicated

^a^μmol mg protein^−1^ min^−1^

## Discussion

Cellulosic ethanol, which is produced from non-edible biomass, has advantages over first-generation biomass ethanol made from food starch [1–3]. Despite its potential practical uses and the construction of several demonstrational or commercial plants, large-scale production of ethanol from cellulose and hemicellulose has not yet been realized. In addition to high cellulase production costs, the cost of cellulosic biomass as an inedible agricultural waste is rising, such that the total production costs for cellulosic ethanol production will likely further increase. Although multiple studies have shown the potential for practical use of XI enzyme expressed by *S. cerevisiae* and engineered strains harboring XI have been developed for production of cellulosic ethanol, the fermentation ability of these strains remains too low for practical applications. Therefore, development of more productive and practical ethanologenic microorganisms that can metabolize not only d-glucose from cellulose but also other sugars, especially d-xylose, from hemicellulose, is needed.

As mentioned above, pioneering studies showed the difficulties in functional expression of bacterial XI in yeast [15–26]. In addition, there are few direct comparisons of XI expression efficiency, catalytic activity and fermentation performance using the same platform and under the same conditions [18]. For commercial production of cellulosic ethanol, one of the most critical issues is the need to identify the most effective XI for glucose/xylose co-fermentation. In this study, we first employed an identical platform and growth conditions to comprehensively evaluate the d-xylose consumption and fermentation performance of strains expressing one of eight previously reported XIs. The control strain SS36 that carried no XI did not grow on YPX_50_ media, but five of eight XI-expressing strains, SS38 (*Pr*XI), SS39 (*Rf*XI), SS40 (*Osp*XI), SS41 (*Psp*XI) and SS42 (*Lp*XI), grew well aerobically, clearly indicating that these five XIs, in particular, *Lp*XI and *Osp*XI, can be functionally expressed in yeast. However, *Lp*XI and *Osp*XI showed quite different traits in terms of co-fermentation. Xylose consumption was dramatically improved in the strain expressing *Lp*XI, but not in the strain with *Osp*XI. These inconsistent results could be attributed to differences in functional expression of XI or its protein folding under aerobic growth and micro-aerobic fermentation conditions, although further investigation is needed to explore these possibilities. Interestingly, the habitat environment of the microorganisms from which XI originated could be responsible for these different behaviors. *Osp*XI and *Psp*XI are genes derived from anaerobic fungi that are part of the ruminal microflora of herbivorous animals. Moreover, previous studies demonstrated that recombinant yeast strains expressing these XIs could consume d-xylose [14, 18, 25, 27, 28]. In this study, we used the same promoter, *HSP12*, for XI expression so that the expression level of each XI should be similar. However, even with similar XI expression levels, the protein lifetime or folding related to XI enzymatic activity could differ between aerobic and micro-aerobic conditions. To the best of our knowledge, the sustainability of XI proteins in yeast under aerobic and micro-aerobic conditions has not yet been examined. However, recent studies focusing on XI protein folding demonstrated that co-expression with *Escherichia coli* (*E. coli*) GroEL could facilitate the functional expression of xylose and arabinose isomerase genes from *E. coli* as well as d-psicose isomerase from *Agrobacterium tumefaciens* in *S. cerevisiae* [29]. Moreover, *Propionibacterium acidipropionici* XI is functional in yeast when co-expressed with the molecular chaperones GroEL and GroES [30]. This approach may facilitate functional expression of XI enzymes in yeast.

To better understand requirements for producing ethanol from cellulosic biomass, the relationship between cell growth and fermentation performance under given fermentation conditions must be considered. Of course, cell growth is a crucial issue for fermentation because the number of cells during fermentation directly affects the rate of sugar consumption and ethanol production as well as d-xylose metabolism. In this study, we used the fermentation medium YPD_85_X_35_ that includes sugar concentrations that reflect those of typical cellulosic biomass hydrolysates [31, 32]. The model medium contains high concentrations of sugars, which are needed for commercial production of cellulosic ethanol, particularly to produce higher concentrations of ethanol (e.g., 50 g/L or more) that allow efficient distillation. The sugar consumption rates integrate the viable cell number and the consumption rate per cell as a function of time. The true catalytic activities of XIs in examination of fermentation are thought to be proportional to the consumption rate of d-xylose per cell. However, because the viable cell number changes over time, this value cannot be determined in real time. Therefore, to avoid differences in growth rates between XI-containing strains, high numbers of cells (OD_A600_ = 20) that prevent further cell proliferation were inoculated for examinations of XI activity in micro-aerobic fermentation. Brat and colleagues [18] previously reported that heterogenous expression of *Lp*XI conferred the ability to metabolize d-xylose on both industrial and laboratory yeast strains. Consistent with our results, they showed that strains containing *Lp*XI had superior d-xylose consumption and growth on media having d-xylose as a sole carbon source, but their study did not determine whether *Lp*XI-expressing strains had the best fermentation performance in micro-aerobic glucose/xylose co-fermentation [18]. In our results, both SS42 (wild-type *Lp*XI, episomally) and SS81 (wild-type *Lp*XI, integrated into the chromosome) consumed 85 g/L d-glucose in 12 h and subsequently consumed 25 g/L d-xylose in 72 h. Such incomplete consumption and remaining d-xylose in the fermentation process could be directly associated with decreased ethanol yield. Our results clearly showed that *Lp*XI has the best potential among the eight XIs tested for ethanol production in micro-aerobic glucose/xylose co-fermentation, but the amount of expressed XI protein and its catalytic activity was still low and requires further improvement for use on a commercial scale.

To improve the catalytic activities of *Lp*XI, we carried out evolutionary molecular engineering using error-prone PCR to introduce one or two amino acid mutations in the coding region. At least 90% of plasmids had several mutations per open reading frame (ORF) of *Lp*XI (2.0 mutations on average), indicating that the in vitro mutagenesis was successful (data not shown). After large-scale selection, we determined the amino acid substitutions for 24 candidates that could grow well on high-concentration d-xylose plates (YPX_80_) and that had mutated *Lp*XI. After eliminating false-positives 11 candidates remained, and of these, two strains, SS82 (*Lp*XI-*T63I/A121A*) and SS92 (*Lp*XI-*V162A*/*T273T/N303T*), consumed nearly all d-glucose and d-xylose within 72 h. Co-fermentation examination using either SS104 (*Lp*XI-*V162A*) or SS105 (*Lp*XI-*N303T*) clearly indicated that V162A was the mutation responsible for the improved performance of SS92 (*Lp*XI-*V162A*/*T273T/N303T*). Meanwhile, SS120 carrying the *Lp*XI double mutation T63I and V162A derived from the different two-mutant clones showed that these mutations produced no synergistic improvement in fermentation performance relative to the single mutants. Biochemical assays showed that the *V*_max_ of *Lp*XI-*T63I*/*V162A* (SS120, 0.107 µmol/mg protein/min) was 1.7-fold higher than that of wild-type *Lp*XI (SS81, 0.064 µmol/mg protein/min), while the *K*_m_ value increased to 37.1 mM, which was higher than that for the singly mutated *Lp*XIs. This result could explain why SS120 (*Lp*XI-*T63I*/*V162A*) did not show improved catalytic activities relative to the singly mutated *Lp*XIs. Furthermore, the yeast strains used in this study had no exogenous d-xylose transporters, such that incorporation of d-xylose was the rate-limiting process and may have contributed to low intracellular concentrations of d-xylose. As such, attempts to lower the XI *K*_m_ could be effective in promoting d-xylose metabolism by *S. cerevisiae* cells. Under our experimental conditions, *Psp*XI had the lowest *K*_m_ among the eight XIs at 19.7 mM, which was similar to the 20 mM reported by Kuyper et al. [14], but lower than other previously reported *K*_m_ values for *Psp*XI that ranged between 49.9 and 87.0 mM [18, 21, 25]. The second-best *K*_m_ measured for this study was for *Osp*XI (27.6 mM), but as mentioned above, both *Psp*XI and *Osp*XI were unsuitable for glucose/xylose co-fermentation. Although the wild-type *Lp*XI *K*_m_ of 45.7 mM was comparatively moderate, it was substantially higher than that of hexokinases for d-glucose (1.0 mM) [33].

To compare the performance of engineered C5C6 yeast harboring XIs in glucose/xylose co-fermentation, we adopted four indices: (i) the time to complete consumption of input sugars (*T*); the consumption rate of d-xylose at (ii) the end of co-fermentation (C_xyl(T)_) and (iii) the halfway point of the fermentation period (C_xyl(T/2)_); and (iv) the ethanol yield at the end of co-fermentation (Y_eth(T)_). In typical glucose/xylose co-fermentation, microbes first consume d-glucose via carbon catabolite repression, such that *T* is the practical time to complete consumption of d-xylose. In this study, the *T* value was defined as about 72 h based on the results for SS92 and SS120. These indices depend on cells in the initial inoculum. The C_xyl72_ value represents the amount of d-xylose consumption in 72 h and in particular, the consumption of d-xylose is represented by the difference between the input and residual sugars, so we measured the precise concentrations of input sugars in the medium for each experiment. C_xyl36_ is an index for the d-xylose consumption rate during the early phase of xylose fermentation and also depends on the time needed for d-glucose consumption. For example, if glucose consumption is delayed, d-xylose consumption should also be delayed as active cells do not increase as well. The theoretical ethanol yields of d-glucose and d-xylose are 0.51 (g/g-input sugars). The best ethanol yield seen for SS82, SS92 and SS120 in this study was 0.44 (g/g-input sugars). In particular, the *Lp*XI-*V162A*/*T273T/N303T* and *Lp*XI-*T63I*/*V162A* mutations were effective for improving the xylose utilization rate. The T63I and V162A substitutions in *Lp*XI generated by error-prone PCR contributed to an increase in the d-xylose consumption rate by 1.64- and 1.11-fold, respectively, over that of wild-type *Lp*XI in micro-aerobic glucose/xylose co-fermentation. These novel mutations provide insight for further improvement of XI enzyme activity to develop C5C6 yeast and facilitate commercial production of cellulosic ethanol.

Currently, the best-performing reported C5C6 yeast strain GS1.11-26, which contains wild-type *Lp*XI for d-xylose metabolism, can consume 36 g/L d-glucose and 35 g/L d-xylose within 12 h and has an ethanol yield of 0.46 [19]. This strain could quickly consume almost all d-xylose in just 13 h in a semi-aerobic glucose/xylose co-fermentation with an initial cell density of 1.3 g-dry cell weight/L [19]. Interestingly, the heterogenous gene *Lp*XI in the GS1.11-26 strain was inserted close to an ARS1529 sequence and was amplified by about ninefold for both chromosome alleles [34]. Thus, the amount or activities of XI enzymes would still be rate-limiting for d-xylose metabolism. The fermentation performance for SS120 is not yet comparable to the GS1.11-26 strain. To develop strains with superior fermentation performance in terms of d-xylose consumption and ethanol production rates, expression of d-xylose metabolism genes must first be optimized.

Selection of a promoter to drive expression of a foreign gene is also critical. In this study, we chose the *S. cerevisiae HSP12* promoter to drive XI expression in all strains. This promoter was selected based on the assumption that co-fermentation environments represent a stressful condition for yeast because micro-aerobic culture conditions restrict energy supplies. An approach to determine a promoter that includes an artificial promoter to drive gene expression during micro-aerobic fermentation may be needed. Wild-type *Lp*XI used in GS1.11-26 was driven by the *PYK2* promoter, a paralog of the *CDC19* pyruvate kinase gene in yeast [19]. Because carbon catabolite repression reduces *PYK2* expression in the presence of glucose [35], *Lp*XI should best be expressed during the d-xylose assimilating phase. Factors affecting promoter activity under fermentation conditions, such as temperature, pH, sugar composition and the presence of fermentation inhibitors as well as the timing of gene expression should be considered.

Regulation of coordinated metabolic flux in the d-xylose metabolic pathway is another important consideration for promoter selection. We recently reported that balanced expression of pentose phosphate pathway (PPP) genes would maximize the d-xylose consumption rates during high-temperature glucose/xylose co-fermentation and that the necessary PPP genes for optimization of d-xylose consumption and ethanol production differ between strains with XI and XR-XDH [36, 37]. Furthermore, as mentioned above, we have not yet optimized d-xylose uptake, which can be facilitated by controlled expression of hexose transporters including *HXT7, HXT5, GAL2, HXT1* and *HXT2* [38]. Several recent studies engineered these hexose/pentose transporters to promote specific uptake of d-xylose in yeast (reviewed in [4]). Using d-xylose media to screen mutant clones made by site-directed mutagenesis, the glucose-insensitive xylose transporters *HXT7*-*N370S* and *GAL2*-*N376F* were identified [39]. This glucose insensitivity is an important property for utilization of d-xylose during glucose/xylose co-fermentation because these transporters cannot incorporate d-xylose in the presence of d-glucose due to the higher affinity for d-glucose compared to d-xylose.

Despite the numerous pioneering studies, whether the XR-XDH pathway or XI pathway is superior for yeast-mediated biomass fermentation remains controversial. We believe that metabolic pathways using XI will have advantages over those using XR-XDH for several reasons. For the XR-XDH pathway, the production of byproducts such as xylitol cannot be avoided and the dependence of XR on NADPH should produce a coenzyme imbalance. On the other hand, XI could surmount these issues as the XI protein can directly convert d-xylose to d-xylulose. Indeed, based on our results and those of previous studies, the amount of xylitol byproduct in glucose/xylose co-fermentation was negligible, resulting in an ethanol yield that was even higher than that for the high-performance XR-XDH strain NAPX37 [40]. In any case, the d-xylose consumption rate for SS120 was still tenfold lower than that for d-glucose. The XR-XDH pathway and the XI pathway both have advantages and disadvantages, and which is superior remains a topic of debate. Nonetheless, to develop strains that can be used for the commercial production of cellulosic ethanol, it will be necessary to increase the consumption rate of d-xylose and ethanol productivity by several fold as well as the ethanol yield.

## Conclusions

Here, we compared the d-xylose metabolism of eight previously reported XIs expressed in the yeast strain IR-2 grown on d-glucose and d-xylose that mimics a typical biomass hydrolysate to determine the most suitable XI for industrial ethanol production. Comparative analyses of transformants harboring these XIs in micro-aerobic fermentation showed that *Lp*XI has the highest d-xylose consumption rate in 72 h. Random mutagenesis of *Lp*XI using error-prone PCR yielded two novel and beneficial mutations (T63I and V162A) that were shown to be responsible for improved catalytic activities relative to wild-type. The strain SS120 expressing *Lp*XI-*T63I/V162A* with deletion of the *GRE3* gene and overexpression of the *XKS1* gene produced a maximum rate of ethanol production of 53.3 g/L ethanol from about 85 g/L d-glucose and 35 g/L d-xylose for 72 h under micro-aerobic fermentations. This strain could assimilate nearly all available d-xylose, in contrast to the control strain SS82 expressing wild-type *Lp*XI that left 10 g/L d-xylose. To our knowledge, the novel *Lp*XI-*T63I/V162A* mutant is a superior d-xylose isomerase that could be used to improve the capacity of industrial *S. cerevisiae* strains to efficiently metabolize d-xylose and produce maximum amounts of bioethanol.

## Methods

### Strains, media and culture conditions

Yeast strains used in this study are listed in Additional file 1: Table S1. *S. cerevisiae* SS29 (*MATα ho::bleMX6 gre3::hphMX6*), a haploid strain derived from diploid IR-2, was used as a common host strain [41, 42]. Yeast cells were grown at 30 °C in YPD medium [10 g/L yeast extract (BD Biosciences, Sparks, MD), 20 g/L Bacto™ peptone (BD Biosciences), 20 g/L d-glucose (Sigma-Aldrich, St. Louis, MO)], YPX_50_ medium [10 g/L yeast extract, 20 g/L Bacto™ peptone, 50 g/L d-xylose (Sigma-Aldrich)], YPX_80_ medium (10 g/L yeast extract, 20 g/L Bacto™ peptone, 80 g/L d-xylose), or YPD_85_X_35_ medium (10 g/L yeast extract, 20 g/L Bacto™ peptone, 85 g/L d-glucose, 35 g/L d-xylose). For solid plates, 20 g/L Bacto™ agar (BD Biosciences) was added to the respective liquid media. For selection of transformants, several antibiotics were added to culture media: G418 (200 µg/mL; Thermo Fisher Scientific/Life Technologies, Carlsbad, CA), Zeocin (400 µg/mL; Thermo Fisher Scientific/Life Technologies), hygromycin B (200 µg/mL; Thermo Fisher Scientific/Life Technologies), and/or Aureobasidin A (0.5 µg/mL; Takara-Bio Inc., Shiga, Japan). *Escherichia coli* strain DH5α (Nippon Gene, Co. Ltd., Tokyo, Japan, or BioDynamics Laboratory Inc., Tokyo, Japan) was used for cloning, plasmid propagation, and construction of a mutated XI library. DH5α cells were grown at 37 °C in Luria–Bertani (LB) broth (BD Biosciences) supplemented with 100 µg/mL ampicillin (Wako Pure Chemical Industries, Ltd., Osaka, Japan). Yeast and bacterial strains were stored at − 80 °C in 15% glycerol.

### Design, synthesis and cloning of XI genes

DNA fragments were amplified by polymerase chain reaction (PCR) with KOD-Plus-Neo DNA polymerase (TOYOBO, Osaka, Japan) using the primers listed in Additional file 12: Table S9. An expression vector pUG35-kan was constructed based on a low copy number expression vector pUG35 (GenBank Acc. No. AF298787) as follows: first, for construction of pUG35-MET25, pUG35 was digested with *Sac*I and *Xba*I, treated with a DNA Blunting Kit (Takara-bio), and self-ligated with T4 DNA Ligase (Takara-Bio). A DNA fragment including multiple cloning sites and the CYC1 terminator of pLTex321sV5H was digested with *Sma*I and *Kpn*I and introduced into the *Sma*I and *Kpn*I sites, resulting in pUG35-MET25-EGFP3 + MCS. The *kan*^*r*^ (G418 resistant) gene expression cassette (*kanMX*) was amplified by PCR using a primer set (Kan exu F *Nde*I and Kan exu R *Kpn*I) containing a *Nde*I or *Kpn*I site and using the pUG6 plasmid as a template [43]. The amplified DNA fragment was digested with *Nde*I and *Kpn*I and introduced into *Nde*I and *Kpn*I sites of pUG35-MET25-EGFP3 + MCS, resulting in pUG35-kan. Eight XIs that were previously demonstrated to be functional upon expression in *S. cerevisiae* were selected: *Piromyces* sp. E2 [14], *Orpinomyces* sp. ukk1 [15–17], *Lachnoclostridium phytofermentans* ISDg (*Lp*XI) [18, 19], *Ruminococcus flavefaciens* 17 [20], *Prevotella ruminicola* TC2-24 [21], *Burkholderia cenocepacia* J2315 [22, 23], *Ruminiclostridium cellulolyticum* H10 [24] and *Streptomyces rubiginosus* [25, 26]. The original nucleotide and amino acid sequences of these XIs were obtained from the National Center for Biotechnology Information (NCBI). The synthetic nucleotide sequences of these XI genes were optimized based on codon usage tables derived from highly expressed genes in *S. cerevisiae* [44] and were synthesized by the GeneArt^®^ gene synthesis service (Thermo Fisher Scientific/Life Technologies). The sequences included *Spe*I and *Kpn*I sites for subcloning. The accession numbers of the codon-optimized nucleotide sequences are *Psp*XI (LC424543), *Osp*XI (LC424542), *Lp*XI (LC424544), *Rf*XI (LC424541), *Pr*XI (LC424540), *Bc*XI (LC424539), *Rc*XI (LC424545) and *Sr*XI (LC424546). These codon-optimized XI genes were amplified by PCR with the specific primer sets *XIopt_F1 and *XIopt_R1 (where the asterisks are specific for the species of XI listed above). The *Xho*I sites were added in the reverse primers *XIopt_R1 for directional cloning. The amplified and purified DNA fragments were digested with *Xho*I and then subcloned into *Sma*I and *Xho*I sites of pLTex321sV5H [45] to add a V5-6xHis tag at the C-terminus of each XI gene for purification of XI proteins. The *Spe*I and *Kpn*I fragments containing the XI gene expression cassette, including the *HSP12* promoter, XI gene, and *CYC1* terminator, were transferred into the *Spe*I and *Kpn*I sites of the low copy vector pUG35-kan. In addition, the d-xylulokinase (*XKS1*) expression cassette including the *PGK1* promoter and *XKS1* derived from *S. cerevisiae* was amplified by PCR with a primer set (TCYC1-PPGK1 F and pUG35-TCYC1 R) and pAUR-XKXDHXR plasmid as a template [46]. The fragment was tandemly subcloned into the *Kpn*I site of pUG35-Kan harboring each XI gene cassette using an In-Fusion^®^ HD Cloning Kit (Takara-Bio) according to the manufacturer’s instructions to generate XI expression plasmids pUG35-kan-p*HSP12*-*XI-t*CYC1*-p*PGK1*-*XKS1*-t*CYC1*. XI expression was driven by the stress-responsive *HSP12* promoter, which can induce expression of downstream genes during the d-xylose consumption phase that occurs after glucose depletion, and the *XKS1* gene was overexpressed under the control of the 3-phosphoglycerate kinase gene (*PGK1*) promoter. All DNA sequences of the constructed plasmids were verified by Sanger sequencing (Eurofins Genomics Inc., Tokyo Japan).

### Growth assays under aerobic cultivation

Yeast cells were pre-cultured in YPD medium supplemented with an appropriate antibiotic until the stationary phase was reached. The cells were then washed once with sterilized water before inoculation into growth assay media. YPX_50_ medium was used for cultivation to determine d-xylose assimilation activity of strains expressing XIs under aerobic conditions. Yeast cells at a final OD_A600_ of 0.1 were inoculated into 100 µL YPX_50_ medium in 96-well microplates. Yeast strain growth was then monitored using an Infinite^®^ 200 PRO microplate reader (Tecan, Männedorf, Switzerland). During 4 days of cultivation with continuous shaking at 30 °C, the absorbance (600 nm) of each well containing individual yeast strain cultures was measured every hour. Growth assays for each strain were performed in triplicate. The specific growth rate *μ* (h^−1^) and its mean ± SD were calculated from the slope of a line fitted to the exponential growth curve.

### Fermentation assays under micro-aerobic cultivation

Fermentation assays were carried out as described previously with slight modifications [36, 37]. In brief, cells were pre-cultured to the stationary phase at 30 °C in 50 mL YPD medium supplemented with an appropriate antibiotic and washed once with YPD_85_X_35_ medium. Cells (initial cell density of 20 OD_A600_ unit) were then inoculated into 70 mL fresh YPD_85_X_35_ media in 100-mL flasks capped with rubber stoppers with inserted syringe needles to release CO_2_. The flasks were also fitted with T-shape stopcocks for ventilation and sampling. During fermentation, 500 µL from each culture medium was collected via the sampling port at specified intervals (0, 1, 3, 6, 12, 24, 36, 48, 60 and 72 h). Samples were stored at − 80 °C until assessment by HPLC. All fermentation assays were performed in triplicate unless otherwise stated. To determine the concentration of sugars and fermentation products such as glucose, xylose, xylitol, glycerol, acetate and ethanol in the culture media, specimens were diluted fivefold with deionized water and analyzed by an HPLC instrument coupled to a refractive index detector (Jasco, Tokyo, Japan) and fitted with an Aminex HPX-87H column and Cation H Refill Guard column (Bio-Rad, Hercules, CA). HPLC assays were performed at 65 °C with 5 mM H_2_SO_4_ buffer as the mobile phase, a flow rate of 0.6 mL/min, and an injection volume of 25 µL.

### Construction of an *Lp*XI mutant library

A library of randomly mutated *Lp*XI genes was generated using the Diversify PCR Random Mutagenesis Kit (Clontech Laboratories, Inc., Mountain View, CA) according to the manufacturer’s instructions to perform error-prone PCR with the primer set oSS62 XI-F_HSP12s and oSS74 XI-Rc from pUG35-kan-p*HSP12*-*Cp*XI-t*CYC1*-p*PGK1*-*XKS1*-t*CYC1*. To produce a mutation rate of 2.7 mutations per 1 kb by error-prone PCR, the following reaction conditions were used: 38 µL PCR-grade water, 5 μL TITANIUM *Taq* Buffer (10×), 2 µL MnSO_4_ (8 mM), 1 μL dGTP (2 mM), 1 μL Diversify dNTP Mix (50×), 1 μL primer mix (10 μM each), 1 μL template DNA (1 ng/μL), and 1 μL TITANIUM *Taq* Polymerase. The purified PCR products were cloned into plasmids to generate a library of more than 10^4^ clones. A linearized vector was prepared using inverse PCR with the primer set oSS63 XI-R_HSP12as and oSS83 06_LpXI-Fc from pUG35-kan-p*HSP12*-*Lp*XI-t*CYC1*-p*PGK1*-*XKS1*-t*CYC1* and the KOD-Plus-NEO DNA polymerase. The randomly mutagenized *Lp*XI genes were fused to the linearized vector using an In-Fusion^®^ HD Cloning Kit (Clontech). An aliquot of the reaction solution was used to transform Giga Competent Cells (DH5α, BioDynamics Laboratory, Inc.) that were then used to amplify a plasmid library harboring mutated *Lp*XI genes. To estimate the mutation rate of the *Lp*XI gene, randomly selected plasmids were purified from *E. coli* transformants in the library using a QIAprep Spin Miniprep kit (QIAGEN) and the plasmid nucleotide sequences were determined by Sanger sequencing.

### Identification of beneficial mutations in *Lp*XI

The plasmid library harboring mutated *Lp*XI was purified from *E. coli* transformants and the purified plasmids were introduced into the host strain SS29. To determine the suitable experimental conditions for selection of yeast transformants harboring mutated *Lp*XIs that had improved enzymatic characteristics, the growth rate of SS42 strains expressing wild-type *Lp*XI on YPX plates having varying d-xylose concentrations (20–100 g/L) was first tested. These assays indicated that 80 g/L d-xylose inhibited growth of the control strain SS42 and thus YPX_80_ plates were used to assess the mutant *Lp*XI library. The total number of transformants was 2.4 × 10^5^, indicating that each variant was examined in 24 yeast transformants on average. After the first selection, plasmids containing mutated *Lp*XI genes were prepared from mature colonies that were then used to re-transform SS29 to exclude naturally occurring mutations that were unique to chromosomal rather than plasmid DNA. To determine the *Lp*XI amino acid substitutions, coding regions in the selected clones were verified by DNA sequencing using four primers, oSS62 (XI-F_HSP12s), oSS74 (XI-Rc) and LpXIopt_Seq_F1, and LpXIopt_Seq_R1. For the final verification, each mutated *Lp*XI and *XKS1* expression cassette was introduced into the chromosome in the SS29 strain by homologous recombination to prevent effects from variation in *Lp*XI gene copy number. The homologous recombination was carried out by first subcloning an *Spe*I/*Sph*I fragment containing the mutated *Lp*XI genes from the plasmid pUG35-kan-p*HSP12*-*Lp*XI-t*CYC1*-p*PGK1*-*XKS1*-t*CYC1* into pAUR101 (Takara Bio). The *Lp*XI gene then integrated into the SS29 strain chromosome at the *AUR1* locus. The ability of the resulting strains harboring mutated *Lp*XI to metabolize d-xylose in co-fermentation in YPD_85_X_35_ medium under micro-aerobic conditions at 30 °C was then examined.

### Construction of artificially mutated *Lp*XI genes by separation and combination of mutations found in the mutant library

Site-directed mutagenesis of *Lp*XI was performed to construct expression plasmids harboring mutated *Lp*XI genes (*Lp*XI-*V162A*, *Lp*XI-*N303T* and *Lp*XI-*T63I*/*V162A*). To introduce the mutations into *Lp*XI, the pAUR-kan-p*HSP12*-*Lp*XI-t*CYC1*-p*PGK1*-*XKS1*-t*CYC1* plasmid was fragmentated by inverse PCR using KOD-Plus-Neo DNA polymerase with oSS106 06_LpXI_V162A and oSS107 06_LpXI_V162Aas as primers. An amplified DNA fragment was self-ligated with T4 DNA Ligase, resulting in the plasmid harboring *Lp*XI-*V162A*. The other mutated *Lp*XIs (*Lp*XI-*N303T* and *Lp*XI-*T63I*/*V162A*) were constructed in the same manner using oSS108 (06_LpXI_N303T) and oSS109 (06_LpXI_N303Tas) or oSS106 (06_LpXI_V162A) and oSS107 (06_LpXI_V162Aas) as primers and the plasmid (pAUR-kan-p*HSP12*-*Lp*XI-t*CYC1*-p*PGK1*-*XKS1*-t*CYC1*). The nucleotide sequences of expression plasmids harboring mutated *Lp*XI genes were validated by Sanger sequencing.

### Measurement of d-xylose isomerase catalytic activity

The catalytic activities of d-xylose isomerase in yeast strains harboring *Lp*XI were examined according to a previously described method [14]. In brief, cell lysates from yeast cells of three independent cultivations after 24 h of fermentation in YPD_85_X_35_ medium were prepared using CelLytic™ Y Cell Lysis Reagent for Yeast Cells (Sigma-Aldrich, St. Louis, MO) and Protease Inhibitor Cocktail for use with fungal and yeast extracts (Sigma-Aldrich) with 0.5-mm zirconium beads. Cell extract protein concentrations were determined using Pierce™ 660 nm Protein Assay Reagent (Thermo Fisher Scientific) according to the manufacturer’s instructions. The catalytic activities of cell lysates were assayed at 30 °C by measuring changes in absorbance at 340 nm using an Infinite^®^ 200 PRO spectrophotometer (Tecan), which reflected decreases in the amount of NADH in 100 µL of reaction mixture containing 100 mM Tris–HCl buffer (pH 8.0), 10 mM MgCl_2_, 0.3 mM β-NADH (Oriental Yeast Co. Ltd., Tokyo, Japan), 2 U sorbitol dehydrogenase (Sigma-Aldrich), and 1 mg/mL-cell lysate. The reaction was started by addition of d-xylose at varying concentrations (10, 50, 100 and 500 mM) as a substrate. Each *K*_m_ and *V*_max_ were determined by the Lineweaver–Burk equation (1/*ν* = (*K*_m_/*V*_max_) × 1/[*S*] + 1/*V*_max_) and a plot of 1/[*S*] for the *x*-axis and 1/*ν* for the *y*-axis with a linear approximation. The molar extinction coefficient of 6.25 (mM^−1^ cm^−1^) at 340 nm for NADH was used to calculate of *V*_max_ (µmol mg protein^−1^ min^−1^). The assay was performed in biological triplicates.

## Additional files

**Additional file 1: Table S1.** *Saccharomyces cerevisiae* strains used in this study.

**Additional file 2: Table S2.** Metabolic profiles of recombinant *S. cerevisiae* strains expressing various XIs in glucose/xylose co-fermentation.

**Additional file 3: Figure S1.** Fermentation performance of strains containing plasmid vector expressing previously reported mutated XIs in glucose/xylose co-fermentation under micro-aerobic conditions. Batch fermentation assays were performed under the same conditions as for Figs. 2, 3, 4. The fermentation properties of seven strains harboring previously reported mutations for *Rf*XI and *Psp*XI are: SS45 (*Rf*XI-*G178A*), SS46 (*Psp*XI-*E15D*), SS47 (*Psp*XI-*C54R*), SS48 (*Psp*XI-*T142S*), SS29 (*Psp*XI-*N370S*), SS50 (*Psp*XI-*C54R, N370S*) and SS51 (*Psp*XI-*E51D, T142S*). These mutated XIs were episomally introduced on low copy number plasmids into the same parental strain, SS29. The dots in the panels represent the concentrations of the following metabolites in a single experiment performed with biological triplicates: glucose (blue), xylose (red), xylitol (yellow green), glycerol (purple), acetate (light blue) and ethanol (orange). The details for metabolite concentrations are shown in Additional file 4: Table S3.

**Additional file 4: Table S3.** Metabolic profiles of recombinant *S. cerevisiae* strains expressing previously characterized *Rf*XI and *Psp*XI mutants in glucose/xylose co-fermentation.

**Additional file 5: Figure S2.** Fermentation performance of eleven clones selected in the second growth assay that contained plasmid vectors with mutated *Lp*XI in glucose/xylose co-fermentation under micro-aerobic conditions. Batch fermentation assays were performed under the same conditions as Figs. 2, 3, 4. The fermentation properties were measured for eleven clones containing plasmid vectors carrying mutated *Lp*XIs generated using error-prone PCR: M6-2, M6-6, M6-7, M6-10, M6-11, M6-13, M6-15, M6-19, M6-20, M6-21 and M6-22. The amino acid substitutions in each clone are shown in Table 2. The dots and error bars in the panels represent the mean concentrations and standard deviations, respectively, of the following metabolites in biological triplicates; glucose (blue), xylose (red), xylitol (yellow green), glycerol (purple), acetate (light blue) and ethanol (orange). The details for metabolite concentrations are shown in Additional file 6: Table S4.

**Additional file 6: Table S4.** Metabolic profiles of recombinant *S. cerevisiae* strains expressing evolved *Lp*XIs from plasmid vectors in glucose/xylose co-fermentation.

**Additional file 7: Table S5.** Metabolic profiles of recombinant *S. cerevisiae* strains expressing mutated *Lp*XIs in glucose/xylose co-fermentation.

**Additional file 8: Figure S3.** Micro-aerobic fermentation of strains expressing mutated XIs on medium containing d-glucose and d-xylose. Batch fermentation assays were performed under the same conditions as for Fig. 2. The fermentation properties were measured for seven strains without improved d-xylose consumption rates: SS84 (*Lp*XI-*K136T/A176T*), SS85 (*Lp*XI-*Y13H/D228V*), SS86 (*Lp*XI-*T273A*), SS87 (*Lp*XI-*D207G*), SS88 (*Lp*XI-*N223I*), SS91 (*Lp*XI-*E114G*) and SS94 (*Lp*XI-*L304S*). These mutated *Lp*XI expression cassettes were introduced into the *AUR1* locus of the SS29 parental strain chromosome. The dots in the panels represent the concentrations of the following metabolites in a single experiment performed in biological triplicate: glucose (blue), xylose (red), xylitol (yellow green), glycerol (purple), acetate (light blue) and ethanol (orange). The details of metabolite concentrations are shown in Additional file 9: Table S6.

**Additional file 9: Table S6.** Metabolic profiles of recombinant *S. cerevisiae* strains expressed mutated *Lp*XIs in a glucose/xylose co-fermentation.

**Additional file 10: Table S7.** Metabolic profiles of recombinant *S. cerevisiae* strains expressed mutated *Lp*XIs in a glucose/xylose co-fermentation.

**Additional file 11: Table S8.** Kinetic analysis of mutated *Lp*XIs

**Additional file 12: Table S9.** Oligonucleotides used in this study.

## Data Availability

All data supporting the conclusions of this article are included within the manuscript and additional files.

## Abbreviations

YPD: yeast extract/peptone/dextrose
YPX: yeast extract/peptone/xylose
LB: Luria–Bertani
OD: optical density
XI: xylose isomerase
XR: xylose reductase
XDH: xylitol dehydrogenase
HPLC: high-performance liquid chromatography
PCR: polymerase chain reaction
HMF: 5-hydroxymethyl-2-furfural
*Osp*XI: xylose isomerase derived from *Orpinomyces* sp. Ukk1
*Lp*XI: xylose isomerase derived from *Lachnoclostridium* (previously known as *Clostridium*) *phytofermentans* ISDg
*Rf*XI: xylose isomerase derived from *Ruminococcus flavefaciens* 17
*Pr*XI: xylose isomerase derived from *Prevotella ruminicola* TC2-24
*Bc*XI: xylose isomerase derived from *Burkholderia cenocepacia* J2315
*Rc*XI: xylose isomerase derived from *Ruminiclostridium* (previously known as *Clostridium*) *cellulolyticum* H10
*Sr*XI: xylose isomerase derived from *Streptomyces rubiginosus*
*XKS1*: xylulokinase derived from *S. cerevisiae*
p*PGK1*: promoter of 3-phosphoglycerate kinase derived from *S. cerevisiae*
p*HSP12*: promoter of heat shock protein 12 derived from *S. cerevisiae*
t*CYC1*: terminator of cytochrome c, isoform 1 derived from *S. cerevisiae*
PPP: pentose phosphate pathway
