# Supplementary material for: Molecular evolutionary engineering of xylose isomerase to improve its catalytic activity and performance of micro-aerobic glucose/xylose co-fermentation in Saccharomyces cerevisiae
Source: Biotechnol Biofuels. 2019 Jun 6;12:139. doi: 10.1186/s13068-019-1474-z (PMC6551904; doi:10.1186/s13068-019-1474-z)
Supplement: Supplementary file 1 — Additional file 1: Table S1. Saccharomyces cerevisiae strains used in this study. [file 13068_2019_1474_MOESM1_ESM.pdf]

Strains	Genotype	Source
IR-2	*Mata/α*	[25]
2a-3-34A	*IR-2 Matα ho::bleMX6*	Lab stock
SS29	*2a-3-34A; gre3::hphMX6*	This study
SS36	*SS29 [pUG35-kan-pHSP12-tCYC1-pPGK1-XKS1-tCYC1]*	This study
SS37	*SS29 [pUG35-kan-pHSP12-BcXI-tCYC1-pPGK1-XKS1-tCYC1]*	This study
SS38	*SS29 [pUG35-kan-pHSP12-PrXI-tCYC1-pPGK1-XKS1-tCYC1]*	This study
SS39	*SS29 [pUG35-kan-pHSP12-RfXI-tCYC1-pPGK1-XKS1-tCYC1]*	This study
SS40	*SS29 [pUG35-kan-pHSP12-OspXI-tCYC1-pPGK1-XKS1-tCYC1]*	This study
SS41	*SS29 [pUG35-kan-pHSP12-PspXI-tCYC1-pPGK1-XKS1-tCYC1]*	This study
SS42	*SS29 [pUG35-kan-pHSP12-LpXI-tCYC1-pPGK1-XKS1-tCYC1]*	This study
SS43	*SS29 [pUG35-kan-pHSP12-RcXI-tCYC1-pPGK1-XKS1-tCYC1]*	This study
SS44	*SS29 [pUG35-kan-pHSP12-SrXI-tCYC1-pPGK1-XKS1-tCYC1]*	This study
SS45	*SS29 [pUG35-kan-pHSP12-RfXI[G179A]-pPGK1-XKS1-tCYC1]*	This study
SS46	*SS29 [pUG35-kan-pHSP12-PspXI[E15D]-tCYC1-pPGK1-XKS1-tCYC1]*	This study
SS47	*SS29 [pUG35-kan-pHSP12-PspXI[C54R]-tCYC1-pPGK1-XKS1-tCYC1]*	This study
SS48	*SS29 [pUG35-kan-pHSP12-PspXI[T142S]-tCYC1-pPGK1-XKS1-tCYC1]*	This study
SS49	*SS29 [pUG35-kan-pHSP12-PspXI[N370S]-tCYC1-pPGK1-XKS1-tCYC1]*	This study
SS50	*SS29 [pUG35-kan-pHSP12-PspXI[C54R,N370S]-tCYC1-pPGK1-XKS1-tCYC1]*	This study
SS51	*SS29 [pUG35-kan-pHSP12-PspXI[E51D,T142S]-tCYC1-pPGK1-XKS1-tCYC1]*	This study
SS81	*SS29 aur1::kanMX6-pHSP12-LpXI-tCYC1-pPGK1-XKS1-CYC1t-AUR1-C*	This study
SS82	*SS29 aur1::kanMX6-pHSP12-LpXI[T63I]-tCYC1-pPGK1-XKS1-tCYC1-AUR1-C*	This study
SS84	*SS29 aur1::kanMX6-pHSP12-LpXI[K136T,A176T]-tCYC1-pPGK1-XKS1-tCYC1-AUR1-C*	This study
SS85	*SS29 aur1::kanMX6-pHSP12-LpXI[Y13H,D228V]-tCYC1-pPGK1-XKS1-tCYC1-AUR1-C*	This study
SS86	*SS29 aur1::kanMX6-pHSP12-LpXI[T273A]-tCYC1-pPGK1-XKS1-tCYC1-AUR1-C*	This study
SS87	*SS29 aur1::kanMX6-pHSP12-LpXI[D207G]-tCYC1-pPGK1-XKS1-tCYC1-AUR1-C*	This study
SS88	*SS29 aur1::kanMX6-pHSP12-LpXI[N223I]-tCYC1-pPGK1-XKS1-tCYC1-AUR1-C*	This study
SS89	*SS29 aur1::kanMX6-pHSP12-LpXI[L78S]-tCYC1-pPGK1-XKS1-tCYC1-AUR1-C*	This study
SS91	*SS29 aur1::kanMX6-pHSP12-LpXI[E114G]-tCYC1-pPGK1-XKS1-tCYC1-AUR1-C*	This study
SS92	*SS29 aur1::kanMX6-pHSP12-LpXI[V162A,N303T]-tCYC1-pPGK1-XKS1-tCYC1-AUR1-C*	This study
SS93	*SS29 aur1::kanMX6-pHSP12-LpXI[R191K,E192K]-tCYC1-pPGK1-XKS1-tCYC1-AUR1-C*	This study
SS94	*SS29 aur1::kanMX6-pHSP12-LpXI[L304S]-tCYC1-pPGK1-XKS1-tCYC1-AUR1-C*	This study
SS104	*SS29 aur1::kanMX6-pHSP12-LpXI[V162A]-tCYC1-pPGK1-XKS1-tCYC1-AUR1-C*	This study
SS105	*SS29 aur1::kanMX6-pHSP12-LpXI[N303T]-tCYC1-pPGK1-XKS1-tCYC1-AUR1-C*	This study
SS120	*SS29 aur1::kanMX6-pHSP12-LpXI[T63I,V162A]-tCYC1-pPGK1-XKS1-tCYC1-AUR1-C*	This study
