# Supplementary material for: Molecular evolutionary engineering of xylose isomerase to improve its catalytic activity and performance of micro-aerobic glucose/xylose co-fermentation in Saccharomyces cerevisiae
Source: Biotechnol Biofuels. 2019 Jun 6;12:139. doi: 10.1186/s13068-019-1474-z (PMC6551904; doi:10.1186/s13068-019-1474-z)
Supplement: Supplementary file 2 — Additional file 2: Table S2. Metabolic profiles of recombinant S. cerevisiae strains expressing various XIs in glucose/xylose co-fermentation. [file 13068_2019_1474_MOESM2_ESM.pdf]

Strains	Time (h)	Glucose	Xylose	Xylitol	Glycerol	Acetate	Ethanol
SS36	Input	83.35 ± 2.09	35.31 ± 0.99	n.d.	n.d.	n.d.	n.d.
	0	81.93 ± 2.24	35.03 ± 1.15	n.d.	n.d.	n.d.	0.44 ± 0.38
	1	74.48 ± 1.68	34.26 ± 0.75	0.19 ± 0.33	1.04 ± 0.22	n.d.	3.48 ± 0.81
	3	53.18 ± 5.31	33.35 ± 0.49	0.21 ± 0.36	2.48 ± 0.28	n.d.	12.34 ± 1.96
	6	2.92 ± 3.66	32.66 ± 1.25	0.53 ± 0.48	4.58 ± 0.41	0.84 ± 0.19	34.95 ± 2.40
	12	n.d.	32.59 ± 0.93	0.77 ± 0.17	4.74 ± 0.23	0.90 ± 0.19	36.12 ± 1.04
	24	n.d.	35.04 ± 1.25	0.90 ± 0.19	5.12 ± 0.33	0.99 ± 0.20	36.99 ± 0.53
	36	n.d.	34.55 ± 1.10	0.99 ± 0.17	5.09 ± 0.31	1.05 ± 0.19	37.73 ± 0.61
	48	n.d.	33.41 ± 0.95	1.07 ± 0.19	4.94 ± 0.33	1.12 ± 0.19	37.79 ± 0.60
	60	n.d.	32.70 ± 1.21	1.16 ± 0.21	4.87 ± 0.35	1.19 ± 0.21	37.41 ± 0.67
	72	n.d.	31.80 ± 1.16	1.24 ± 0.21	4.77 ± 0.32	1.26 ± 0.21	37.25 ± 1.02
SS37	Input	83.35 ± 2.09	35.31 ± 0.99	n.d.	n.d.	n.d.	n.d.
	0	82.32 ± 2.21	35.11 ± 1.24	n.d.	n.d.	n.d.	0.20 ± 0.35
	1	75.15 ± 1.75	34.35 ± 1.28	0.19 ± 0.33	1.01 ± 0.27	n.d.	3.26 ± 0.86
	3	54.87 ± 4.56	33.52 ± 1.66	0.41 ± 0.37	2.43 ± 0.34	n.d.	11.72 ± 1.56
	6	3.55 ± 4.54	33.19 ± 1.25	0.74 ± 0.19	4.67 ± 0.46	0.83 ± 0.20	35.03 ± 2.69
	12	n.d.	33.41 ± 0.63	0.76 ± 0.20	4.86 ± 0.30	0.90 ± 0.18	36.69 ± 1.61
	24	n.d.	34.77 ± 0.75	0.90 ± 0.21	5.08 ± 0.31	0.98 ± 0.17	37.09 ± 0.30
	36	n.d.	34.55 ± 0.80	0.99 ± 0.22	5.07 ± 0.37	1.06 ± 0.18	38.06 ± 0.19
	48	n.d.	33.26 ± 0.82	1.05 ± 0.22	4.93 ± 0.35	1.13 ± 0.18	37.56 ± 0.86
	60	n.d.	33.10 ± 0.50	1.15 ± 0.22	4.90 ± 0.30	1.21 ± 0.18	37.55 ± 1.19
	72	n.d.	32.26 ± 0.85	1.21 ± 0.24	4.82 ± 0.35	1.29 ± 0.19	37.60 ± 0.88
SS38	Input	83.35 ± 2.09	35.31 ± 0.99	n.d.	n.d.	n.d.	n.d.
	0	81.83 ± 1.52	34.97 ± 0.93	n.d.	n.d.	n.d.	0.21 ± 0.36
	1	73.95 ± 1.72	34.18 ± 1.15	0.19 ± 0.33	1.10 ± 0.25	n.d.	3.66 ± 0.76
	3	52.23 ± 4.39	33.44 ± 0.94	0.39 ± 0.34	2.62 ± 0.27	0.46 ± 0.44	12.88 ± 1.44
	6	1.16 ± 1.35	31.96 ± 1.20	0.73 ± 0.18	4.71 ± 0.36	0.87 ± 0.19	36.18 ± 1.36
	12	n.d.	30.48 ± 0.94	0.80 ± 0.17	4.83 ± 0.29	0.91 ± 0.19	37.32 ± 0.68

	24	n.d.	30.75 ± 0.52	1.03 ± 0.2	5.27 ± 0.27	1.00 ± 0.19	38.88 ± 0.77
	36	n.d.	28.52 ± 0.31	1.22 ± 0.21	5.21 ± 0.27	1.06 ± 0.18	39.92 ± 0.93
	48	n.d.	26.62 ± 0.32	1.40 ± 0.22	5.16 ± 0.25	1.12 ± 0.19	40.51 ± 1.07
	60	n.d.	24.62 ± 0.63	1.57 ± 0.22	5.12 ± 0.29	1.19 ± 0.19	41.13 ± 1.26
	72	n.d.	22.85 ± 0.76	1.74 ± 0.23	5.11 ± 0.31	1.25 ± 0.20	41.52 ± 1.03
SS39	Input	83.35 ± 2.09	35.31 ± 0.99	n.d.	n.d.	n.d.	n.d.
	0	81.55 ± 2.66	34.80 ± 1.41	n.d.	n.d.	n.d.	0.19 ± 0.33
	1	73.78 ± 4.10	33.82 ± 1.41	0.18 ± 0.32	1.02 ± 0.20	n.d.	3.37 ± 0.70
	3	51.57 ± 6.41	32.63 ± 1.62	0.4 ± 0.35	2.50 ± 0.28	0.28 ± 0.49	12.43 ± 1.62
	6	2.71 ± 2.48	33.25 ± 2.26	0.76 ± 0.15	4.87 ± 0.44	0.89 ± 0.24	35.77 ± 1.55
	12	n.d.	29.41 ± 2.03	0.80 ± 0.15	4.87 ± 0.33	0.91 ± 0.22	37.83 ± 0.96
	24	n.d.	29.10 ± 1.27	1.02 ± 0.13	5.43 ± 0.20	1.01 ± 0.22	39.95 ± 0.39
	36	n.d.	25.68 ± 1.26	1.18 ± 0.14	5.38 ± 0.25	1.06 ± 0.22	41.37 ± 0.77
	48	n.d.	22.97 ± 1.49	1.30 ± 0.18	5.41 ± 0.30	1.11 ± 0.23	42.48 ± 1.73
	60	n.d.	20.10 ± 1.43	1.44 ± 0.16	5.32 ± 0.29	1.17 ± 0.23	42.70 ± 0.90
	72	n.d.	17.96 ± 1.52	1.59 ± 0.13	5.33 ± 0.25	1.23 ± 0.23	43.86 ± 0.80
SS40	Input	83.35 ± 2.09	35.31 ± 0.99	n.d.	n.d.	n.d.	n.d.
	0	83.31 ± 1.53	35.59 ± 0.97	n.d.	n.d.	n.d.	0.19 ± 0.33
	1	72.66 ± 2.29	33.35 ± 1.01	0.18 ± 0.32	1.01 ± 0.23	n.d.	3.42 ± 0.69
	3	51.13 ± 4.25	32.65 ± 1.25	0.39 ± 0.34	2.51 ± 0.18	0.28 ± 0.49	12.80 ± 0.97
	6	1.07 ± 1.07	31.76 ± 1.37	0.72 ± 0.17	4.56 ± 0.27	0.83 ± 0.18	36.00 ± 1.15
	12	n.d.	29.81 ± 1.19	0.77 ± 0.18	4.61 ± 0.23	0.89 ± 0.20	37.24 ± 0.91
	24	n.d.	30.77 ± 1.68	0.98 ± 0.18	5.12 ± 0.19	0.98 ± 0.21	38.95 ± 0.52
	36	n.d.	28.89 ± 1.77	1.11 ± 0.16	5.16 ± 0.15	1.04 ± 0.21	40.39 ± 0.82
	48	n.d.	26.17 ± 2.28	1.25 ± 0.20	5.05 ± 0.21	1.08 ± 0.21	40.66 ± 0.95
	60	n.d.	24.36 ± 3.17	1.41 ± 0.22	5.07 ± 0.22	1.16 ± 0.24	41.48 ± 1.20
	72	n.d.	22.72 ± 3.40	1.58 ± 0.25	5.04 ± 0.39	1.22 ± 0.23	41.24 ± 1.19
SS41	Input	83.35 ± 2.09	35.31 ± 0.99	n.d.	n.d.	n.d.	n.d.
	0	80.81 ± 1.82	34.50 ± 1.06	n.d.	n.d.	n.d.	n.d.
	1	71.86 ± 2.23	33.31 ± 1.49	0.19 ± 0.33	0.76 ± 0.71	n.d.	3.74 ± 0.81
	3	49.61 ± 4.13	32.98 ± 2.24	0.43 ± 0.39	2.59 ± 0.46	0.29 ± 0.51	13.51 ± 1.72

	6	1.26 ± 1.29	32.79 ± 1.09	0.72 ± 0.20	4.63 ± 0.36	0.83 ± 0.19	35.77 ± 0.84
	12	n.d.	31.11 ± 0.93	0.74 ± 0.21	4.54 ± 0.35	0.89 ± 0.18	36.32 ± 1.02
	24	n.d.	33.52 ± 0.93	0.93 ± 0.20	4.96 ± 0.37	0.96 ± 0.17	37.59 ± 0.71
	36	n.d.	32.80 ± 0.45	1.04 ± 0.20	4.91 ± 0.32	1.03 ± 0.17	38.33 ± 0.24
	48	n.d.	32.12 ± 1.51	1.17 ± 0.22	4.90 ± 0.45	1.09 ± 0.18	37.78 ± 0.85
	60	n.d.	30.52 ± 1.21	1.24 ± 0.23	4.73 ± 0.37	1.16 ± 0.18	38.03 ± 1.04
	72	n.d.	30.34 ± 2.13	1.38 ± 0.27	4.78 ± 0.54	1.23 ± 0.18	37.76 ± 0.93
SS42	Input	83.35 ± 2.09	35.31 ± 0.99	n.d.	n.d.	n.d.	n.d.
	0	81.71 ± 0.87	34.79 ± 0.52	n.d.	n.d.	n.d.	0.19 ± 0.34
	1	72.41 ± 3.12	33.48 ± 1.45	0.19 ± 0.34	1.07 ± 0.29	n.d.	3.68 ± 1.00
	3	49.06 ± 5.20	32.70 ± 1.46	0.24 ± 0.42	2.69 ± 0.41	0.29 ± 0.51	13.94 ± 2.01
	6	1.00 ± 1.31	31.05 ± 0.59	0.72 ± 0.11	4.70 ± 0.31	0.84 ± 0.19	36.74 ± 1.39
	12	n.d.	25.47 ± 1.05	0.83 ± 0.18	4.73 ± 0.31	0.89 ± 0.21	38.91 ± 1.18
	24	n.d.	23.60 ± 0.73	1.13 ± 0.16	5.44 ± 0.36	0.98 ± 0.21	42.03 ± 0.77
	36	n.d.	19.10 ± 0.68	1.35 ± 0.18	5.44 ± 0.36	1.01 ± 0.21	43.72 ± 0.74
	48	n.d.	15.46 ± 0.53	1.52 ± 0.17	5.39 ± 0.28	1.05 ± 0.21	45.58 ± 1.23
	60	n.d.	12.51 ± 1.07	1.68 ± 0.20	5.31 ± 0.31	1.09 ± 0.22	46.76 ± 0.71
	72	n.d.	9.96 ± 1.08	1.80 ± 0.21	5.33 ± 0.34	1.13 ± 0.23	47.62 ± 1.24
SS43	Input	83.35 ± 2.09	35.31 ± 0.99	n.d.	n.d.	n.d.	n.d.
	0	81.41 ± 2.03	34.61 ± 1.10	n.d.	n.d.	n.d.	n.d.
	1	74.24 ± 2.00	33.84 ± 0.75	0.18 ± 0.31	0.97 ± 0.20	n.d.	3.17 ± 0.73
	3	55.07 ± 4.68	33.27 ± 1.49	0.22 ± 0.38	2.32 ± 0.32	0.45 ± 0.43	11.23 ± 1.72
	6	5.36 ± 6.52	32.60 ± 1.04	0.65 ± 0.12	4.38 ± 0.36	0.88 ± 0.21	34.06 ± 3.20
	12	n.d.	32.24 ± 1.46	0.71 ± 0.18	4.55 ± 0.33	0.94 ± 0.21	36.72 ± 1.30
	24	n.d.	34.43 ± 0.82	0.86 ± 0.16	4.89 ± 0.26	1.02 ± 0.19	37.33 ± 0.64
	36	n.d.	34.26 ± 0.51	0.96 ± 0.16	4.88 ± 0.22	1.11 ± 0.19	38.01 ± 0.64
	48	n.d.	32.91 ± 1.10	1.02 ± 0.22	4.73 ± 0.28	1.18 ± 0.21	37.52 ± 0.81
	60	n.d.	32.39 ± 1.21	1.11 ± 0.24	4.67 ± 0.33	1.27 ± 0.22	37.27 ± 0.65
	72	n.d.	32.20 ± 1.13	1.20 ± 0.26	4.66 ± 0.19	1.35 ± 0.24	37.02 ± 0.90
SS44	Input	83.35 ± 2.09	35.31 ± 0.99	n.d.	n.d.	n.d.	n.d.
	0	81.81 ± 0.91	34.83 ± 0.69	n.d.	n.d.	n.d.	n.d.

1	72.30 ± 1.14	33.01 ± 0.31	0.19 ± 0.32	0.99 ± 0.23	n.d.	3.20 ± 0.72
3	53.96 ± 3.70	32.72 ± 1.41	0.23 ± 0.40	2.39 ± 0.26	n.d.	11.52 ± 1.43
6	3.34 ± 4.18	31.96 ± 0.89	0.72 ± 0.19	4.55 ± 0.36	0.87 ± 0.18	34.43 ± 2.23
12	n.d.	31.92 ± 1.03	0.75 ± 0.18	4.70 ± 0.26	0.91 ± 0.17	35.77 ± 0.53
24	n.d.	34.41 ± 1.03	0.91 ± 0.17	5.09 ± 0.30	1.00 ± 0.16	37.12 ± 0.75
36	n.d.	33.58 ± 0.79	0.99 ± 0.20	4.99 ± 0.29	1.06 ± 0.17	37.05 ± 0.52
48	n.d.	32.61 ± 0.99	1.06 ± 0.22	4.86 ± 0.33	1.13 ± 0.18	37.16 ± 0.68
60	n.d.	32.23 ± 0.78	1.15 ± 0.23	4.87 ± 0.31	1.21 ± 0.20	37.25 ± 0.73
72	n.d.	32.03 ± 1.10	1.24 ± 0.26	4.88 ± 0.32	1.29 ± 0.18	36.86 ± 0.74
