# Supplementary figures and images for: Molecular evolutionary engineering of xylose isomerase to improve its catalytic activity and performance of micro-aerobic glucose/xylose co-fermentation in Saccharomyces cerevisiae
Source: Biotechnol Biofuels. 2019 Jun 6;12:139. doi: 10.1186/s13068-019-1474-z (PMC6551904; doi:10.1186/s13068-019-1474-z)

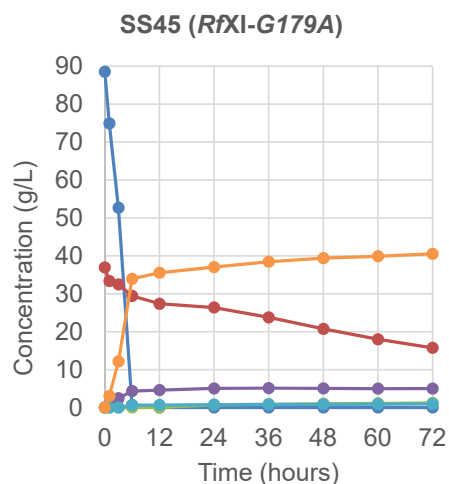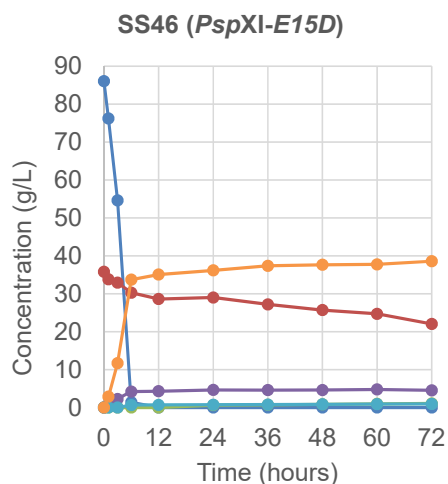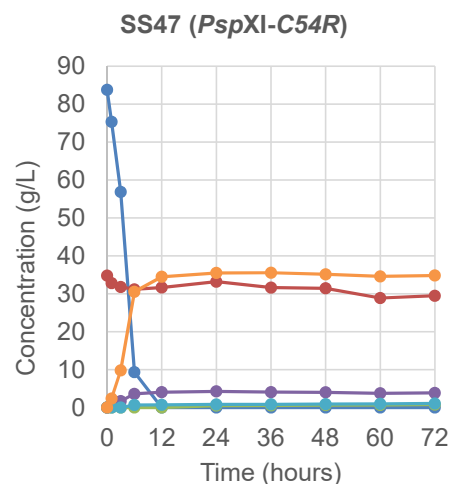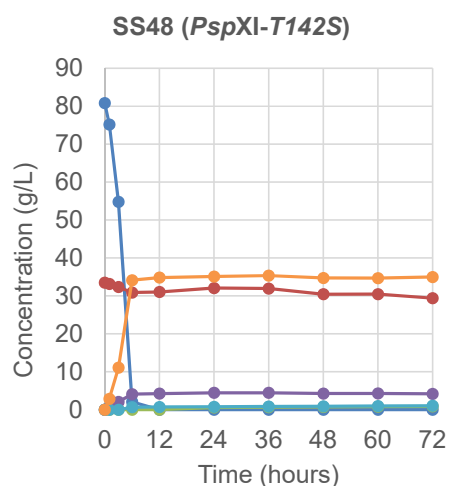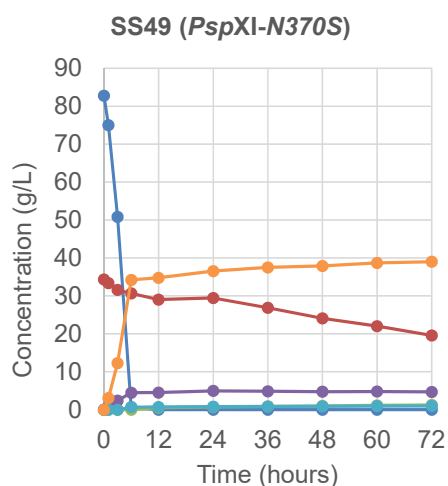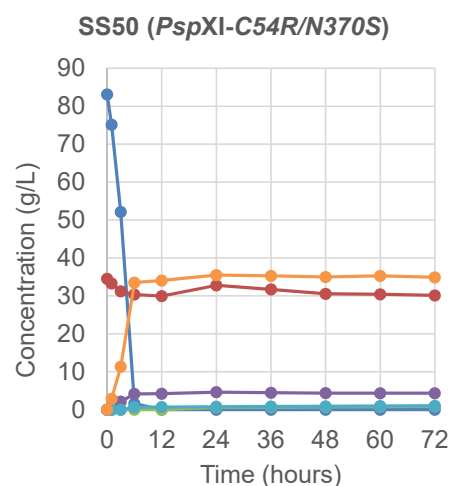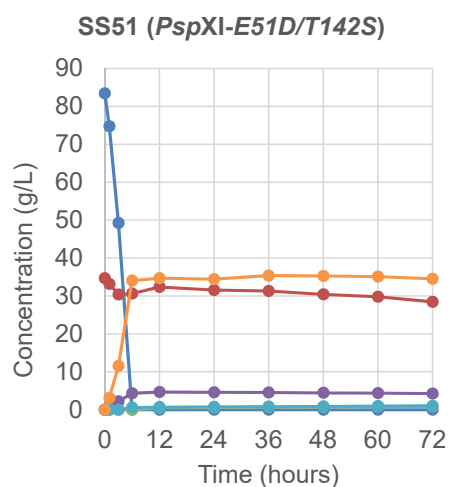

Supplement: Supplementary file 3 — Additional file 3: Figure S1. Fermentation performance of strains containing plasmid vector expressing previously reported mutated XIs in glucose/xylose co-fermentation under micro-aerobic conditions. Batch fermentation assays were performed under the same conditions as for Figs. 2, 3, 4. The fermentation properties of seven strains harboring previously reported mutations for RfXI and PspXI are: SS45 (RfXI-G178A), SS46 (PspXI-E15D), SS47 (PspXI-C54R), SS48 (PspXI-T142S), SS29 (PspXI-N370S), SS50 (PspXI-C54R, N370S) and SS51 (PspXI-E51D, T142S). These mutated XIs were episomally introduced on low copy number plasmids into the same parental strain, SS29. The dots in the panels represent the concentrations of the following metabolites in a single experiment performed with biological triplicates: glucose (blue), xylose (red), xylitol (yellow green), glycerol (purple), acetate (light blue) and ethanol (orange). The details for metabolite concentrations are shown in Additional file 4: Table S3. [file 13068_2019_1474_MOESM3_ESM.pdf]

**M6-2**

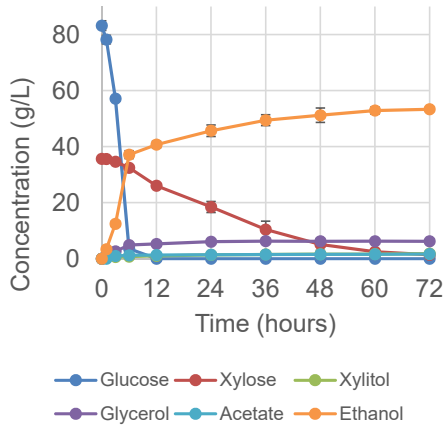

**M6-6**

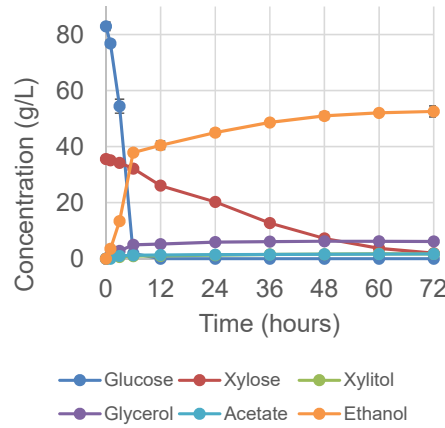

**M6-7**

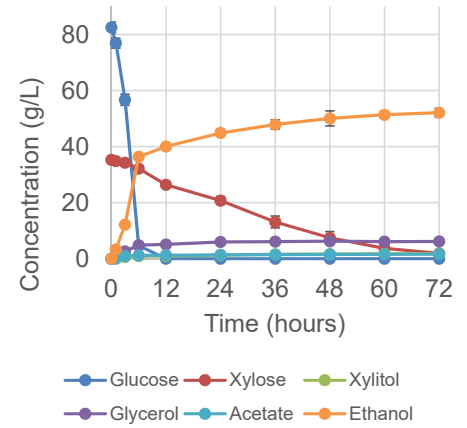

**M6-10**

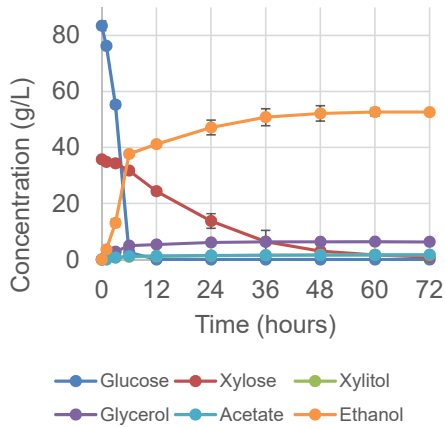

**M6-11**

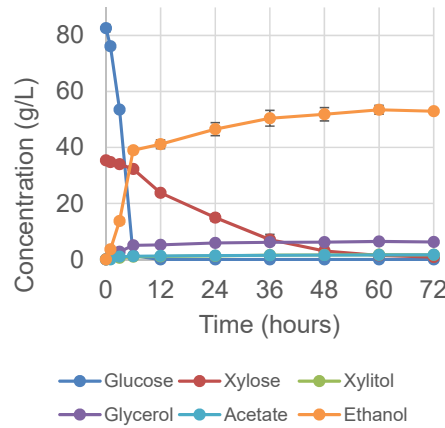

M6-13

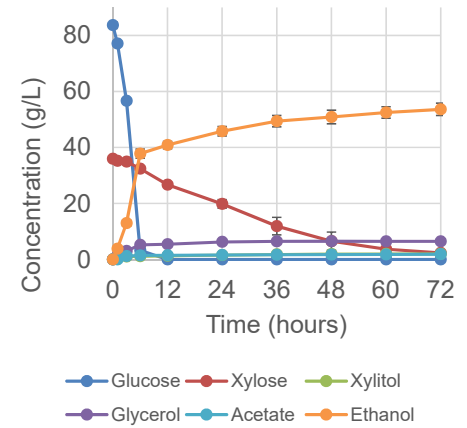

**M6-15**

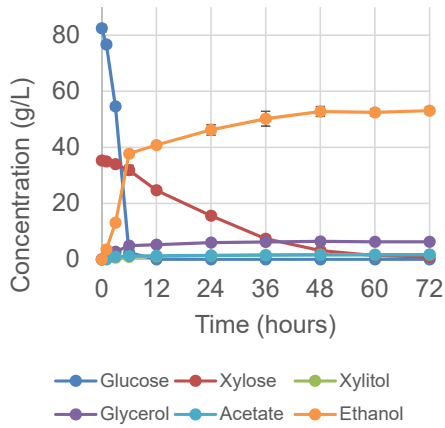

**M6-19**

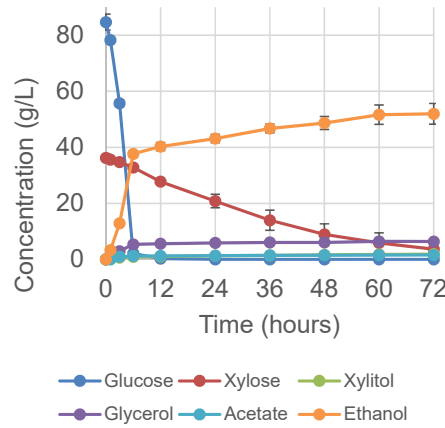

M6-20

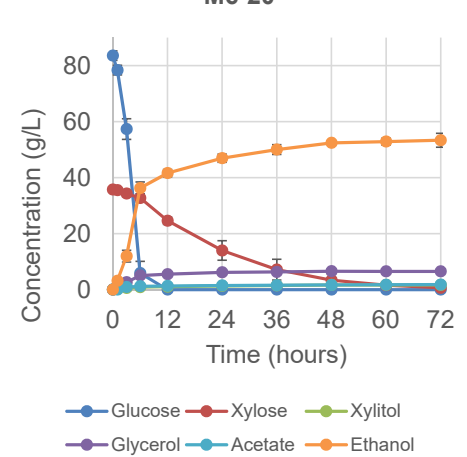

M6-21

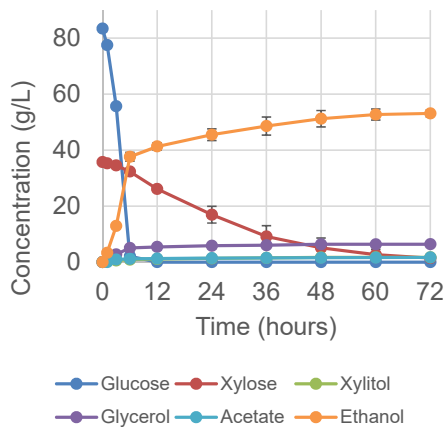

**M6-22**

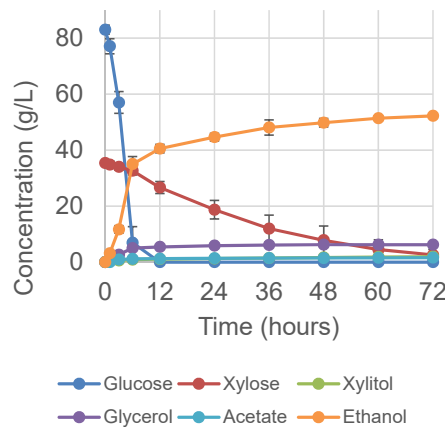

Supplement: Supplementary file 5 — Additional file 5: Figure S2. Fermentation performance of eleven clones selected in the second growth assay that contained plasmid vectors with mutated LpXI in glucose/xylose co-fermentation under micro-aerobic conditions. Batch fermentation assays were performed under the same conditions as Figs. 2, 3, 4. The fermentation properties were measured for eleven clones containing plasmid vectors carrying mutated LpXIs generated using error-prone PCR: M6-2, M6-6, M6-7, M6-10, M6-11, M6-13, M6-15, M6-19, M6-20, M6-21 and M6-22. The amino acid substitutions in each clone are shown in Table 2. The dots and error bars in the panels represent the mean concentrations and standard deviations, respectively, of the following metabolites in biological triplicates; glucose (blue), xylose (red), xylitol (yellow green), glycerol (purple), acetate (light blue) and ethanol (orange). The details for metabolite concentrations are shown in Additional file 6: Table S4. [file 13068_2019_1474_MOESM5_ESM.pdf]

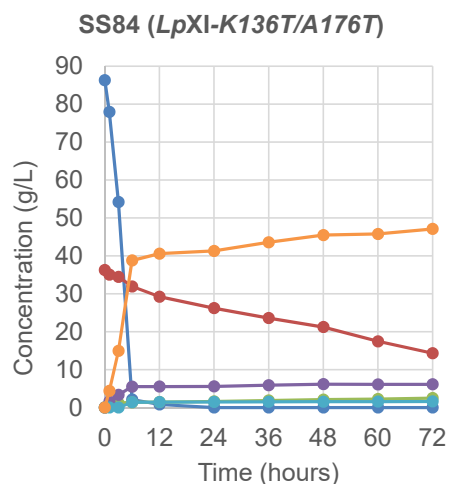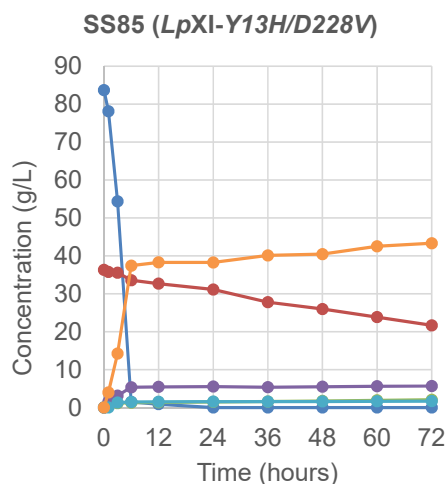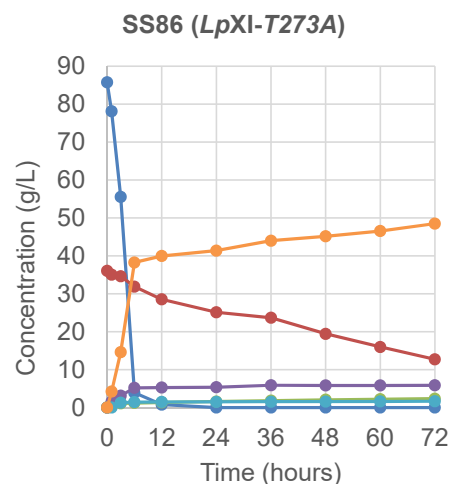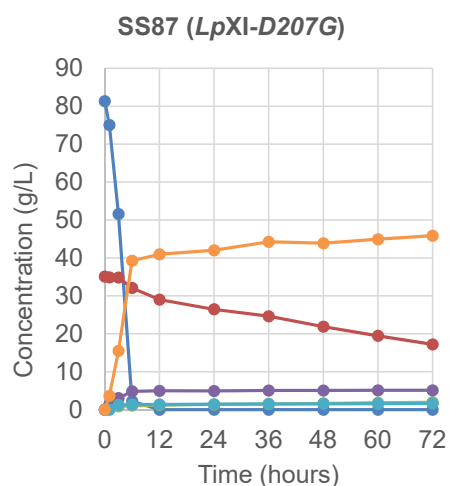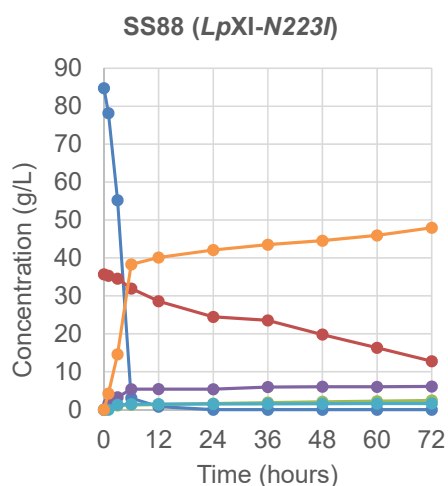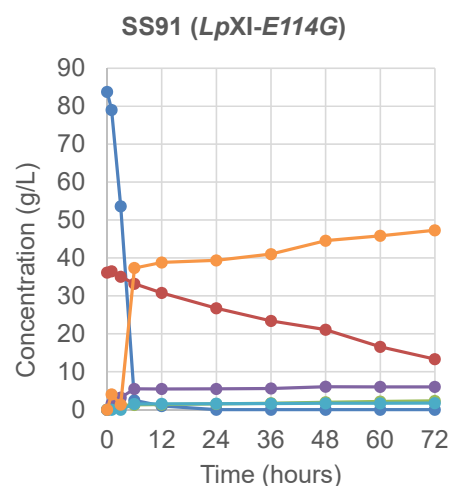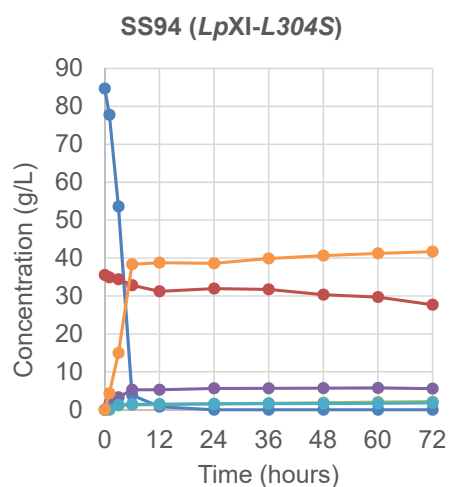

Supplement: Supplementary file 8 — Additional file 8: Figure S3. Micro-aerobic fermentation of strains expressing mutated XIs on medium containing d-glucose and d-xylose. Batch fermentation assays were performed under the same conditions as for Fig. 2. The fermentation properties were measured for seven strains without improved d-xylose consumption rates: SS84 (LpXI-K136T/A176T), SS85 (LpXI-Y13H/D228V), SS86 (LpXI-T273A), SS87 (LpXI-D207G), SS88 (LpXI-N223I), SS91 (LpXI-E114G) and SS94 (LpXI-L304S). These mutated LpXI expression cassettes were introduced into the AUR1 locus of the SS29 parental strain chromosome. The dots in the panels represent the concentrations of the following metabolites in a single experiment performed in biological triplicate: glucose (blue), xylose (red), xylitol (yellow green), glycerol (purple), acetate (light blue) and ethanol (orange). The details of metabolite concentrations are shown in Additional file 9: Table S6. [file 13068_2019_1474_MOESM8_ESM.pdf]
