# Supplementary material for: Molecular evolutionary engineering of xylose isomerase to improve its catalytic activity and performance of micro-aerobic glucose/xylose co-fermentation in Saccharomyces cerevisiae
Source: Biotechnol Biofuels. 2019 Jun 6;12:139. doi: 10.1186/s13068-019-1474-z (PMC6551904; doi:10.1186/s13068-019-1474-z)
Supplement: Supplementary file 4 — Additional file 4: Table S3. Metabolic profiles of recombinant S. cerevisiae strains expressing previously characterized RfXI and PspXI mutants in glucose/xylose co-fermentation. [file 13068_2019_1474_MOESM4_ESM.pdf]

Strain	Time (h)	Glucose	Xylose	Xylitol	Glycerol	Acetate	Ethanol
SS45	Input	83.04	34.23	n.d.	n.d.	n.d.	n.d.
	0	88.54	36.93	n.d.	n.d.	n.d.	n.d.
	1	74.93	33.37	n.d.	0.85	n.d.	3.04
	3	52.67	32.45	n.d.	2.45	n.d.	12.18
	6	0.69	29.43	n.d.	4.36	0.62	33.96
	12	n.d.	27.38	n.d.	4.61	0.70	35.53
	24	n.d.	26.39	0.74	5.05	0.77	37.04
	36	n.d.	23.79	0.89	5.12	0.82	38.44
	48	n.d.	20.75	1.02	5.05	0.86	39.41
	60	n.d.	18.00	1.12	5.00	0.90	39.88
	72	n.d.	15.75	1.24	5.01	0.94	40.54
SS46	Input	83.04	34.23	n.d.	n.d.	n.d.	n.d.
	0	86.04	35.82	n.d.	n.d.	n.d.	n.d.
	1	76.23	33.81	n.d.	0.79	n.d.	2.88
	3	54.61	32.95	n.d.	2.28	n.d.	11.70
	6	1.40	30.28	n.d.	4.20	0.66	33.73
	12	n.d.	28.63	n.d.	4.31	0.70	35.08
	24	n.d.	29.03	0.64	4.66	0.75	36.17
	36	n.d.	27.20	0.74	4.61	0.80	37.38
	48	n.d.	25.71	0.84	4.63	0.85	37.65
	60	n.d.	24.72	0.97	4.77	0.92	37.76
	72	n.d.	22.04	1.08	4.55	0.97	38.58
SS47	Input	83.04	34.23	n.d.	n.d.	n.d.	n.d.
	0	83.76	34.78	n.d.	n.d.	n.d.	n.d.
	1	75.33	32.82	n.d.	0.63	n.d.	2.40
	3	56.87	31.81	n.d.	1.71	n.d.	9.80
	6	9.33	31.17	n.d.	3.62	0.68	30.55
	12	n.d.	31.65	n.d.	4.05	0.73	34.48

	24	n.d.	33.21	0.51	4.27	0.83	35.51
	36	n.d.	31.64	0.56	4.09	0.86	35.55
	48	n.d.	31.44	0.65	4.03	0.93	35.15
	60	n.d.	28.88	0.68	3.78	0.99	34.59
	72	n.d.	29.48	0.79	3.88	1.08	34.83
SS48	Input	83.04	34.23	n.d.	n.d.	n.d.	n.d.
	0	80.84	33.48	0.00	0.00	0.00	0.00
	1	75.18	33.16	0.00	0.74	0.00	2.86
	3	54.79	32.34	0.00	2.07	0.00	11.08
	6	2.07	30.90	0.00	4.10	0.66	34.15
	12	0.00	31.04	0.00	4.25	0.71	34.82
	24	0.00	32.07	0.57	4.45	0.77	35.10
	36	0.00	31.94	0.65	4.44	0.84	35.35
	48	0.00	30.44	0.71	4.27	0.89	34.73
	60	0.00	30.46	0.80	4.29	0.96	34.67
	72	0.00	29.40	0.86	4.17	1.03	34.99
SS49	Input	83.04	34.23	n.d.	n.d.	n.d.	n.d.
	0	82.79	34.33	0.00	0.00	0.00	0.00
	1	75.02	33.36	0.00	0.86	0.00	3.08
	3	50.85	31.58	0.00	2.39	0.00	12.29
	6	0.67	30.67	0.00	4.47	0.64	34.21
	12	0.00	29.03	0.52	4.52	0.71	34.75
	24	0.00	29.44	0.72	4.96	0.80	36.54
	36	0.00	26.88	0.86	4.84	0.84	37.52
	48	0.00	24.09	0.99	4.75	0.89	37.89
	60	0.00	22.03	1.15	4.79	0.95	38.68
	72	0.00	19.57	1.25	4.68	0.98	39.01
SS50	Input	83.04	34.23	n.d.	n.d.	n.d.	n.d.
	0	83.14	34.50	0.00	0.00	0.00	0.00
	1	75.16	33.28	0.00	0.79	0.00	2.84
	3	52.15	31.23	0.00	2.13	0.00	11.35

	6	1.61	30.35	0.00	4.16	0.65	33.50
	12	0.00	29.95	0.00	4.21	0.69	34.06
	24	0.00	32.78	0.62	4.62	0.78	35.49
	36	0.00	31.71	0.65	4.46	0.83	35.26
	48	0.00	30.54	0.71	4.36	0.88	34.99
	60	0.00	30.42	0.79	4.33	0.97	35.27
	72	0.00	30.14	0.87	4.35	1.02	34.90
SS51	Input	83.04	34.23	n.d.	n.d.	n.d.	n.d.
	0	83.41	34.67	0.00	0.00	0.00	0.00
	1	74.72	33.15	0.00	0.82	0.00	3.14
	3	49.23	30.40	0.00	2.22	0.00	11.57
	6	0.00	30.66	0.00	4.35	0.60	34.07
	12	0.00	32.38	0.56	4.67	0.70	34.69
	24	0.00	31.57	0.62	4.61	0.76	34.44
	36	0.00	31.33	0.73	4.57	0.83	35.38
	48	0.00	30.44	0.77	4.45	0.89	35.26
	60	0.00	29.77	0.85	4.40	0.96	35.10
	72	0.00	28.45	0.91	4.28	1.03	34.51
