# Supplementary material for: Molecular evolutionary engineering of xylose isomerase to improve its catalytic activity and performance of micro-aerobic glucose/xylose co-fermentation in Saccharomyces cerevisiae
Source: Biotechnol Biofuels. 2019 Jun 6;12:139. doi: 10.1186/s13068-019-1474-z (PMC6551904; doi:10.1186/s13068-019-1474-z)
Supplement: Supplementary file 6 — Additional file 6: Table S4. Metabolic profiles of recombinant S. cerevisiae strains expressing evolved LpXIs from plasmid vectors in glucose/xylose co-fermentation. [file 13068_2019_1474_MOESM6_ESM.pdf]

Strain	Time (h)	Glucose	Xylose	Xylitol	Glycerol	Acetate	Ethanol
M6-2	Input	84.71 ± 0.98	36.01 ± 0.52	n.d.	n.d.	n.d.	n.d.
	0	83.22 ± 1.70	35.67 ± 1.01	n.d.	n.d.	n.d.	n.d.
	1	78.21 ± 1.52	35.60 ± 0.72	0.56 ± 0.57	1.17 ± 0.44	n.d.	3.36 ± 0.64
	3	57.17 ± 0.18	34.64 ± 0.54	0.61 ± 0.63	2.62 ± 0.52	0.95 ± 0.39	12.48 ± 1.01
	6	3.55 ± 0.77	32.45 ± 0.12	0.72 ± 0.72	4.88 ± 0.56	1.21 ± 0.37	37.13 ± 1.40
	12	n.d.	26.04 ± 0.20	1.02 ± 0.51	5.27 ± 0.61	1.28 ± 0.38	40.73 ± 1.21
	24	n.d.	18.46 ± 1.99	1.35 ± 0.47	6.07 ± 0.61	1.42 ± 0.37	45.69 ± 2.05
	36	n.d.	10.42 ± 2.98	1.55 ± 0.47	6.26 ± 0.63	1.50 ± 0.36	49.43 ± 1.94
	48	n.d.	5.12 ± 2.11	1.59 ± 0.54	6.21 ± 0.70	1.53 ± 0.38	51.21 ± 2.57
	60	n.d.	2.48 ± 1.11	1.65 ± 0.48	6.23 ± 0.59	1.59 ± 0.38	52.90 ± 1.26
	72	n.d.	1.39 ± 0.42	1.81 ± 0.31	6.21 ± 0.54	1.66 ± 0.39	53.35 ± 0.82
M6-4	Input	84.61 ± 1.14	35.86 ± 0.78	n.d.	n.d.	n.d.	n.d.
	0	82.70 ± 1.80	35.35 ± 1.11	n.d.	n.d.	n.d.	n.d.
	1	76.75 ± 1.73	34.83 ± 1.11	0.55 ± 0.55	1.18 ± 0.42	n.d.	3.40 ± 0.70
	3	55.56 ± 0.34	34.22 ± 1.20	0.61 ± 0.62	2.71 ± 0.44	0.77 ± 0.72	12.73 ± 1.46
	6	3.16 ± 1.23	32.19 ± 0.74	0.90 ± 0.42	4.88 ± 0.40	1.17 ± 0.41	36.81 ± 2.19
	12	n.d.	27.41 ± 0.67	1.00 ± 0.45	5.16 ± 0.37	1.26 ± 0.44	40.06 ± 1.57
	24	n.d.	22.04 ± 0.31	1.23 ± 0.36	5.93 ± 0.36	1.39 ± 0.45	43.79 ± 1.99
	36	n.d.	15.40 ± 0.95	1.48 ± 0.47	6.08 ± 0.37	1.46 ± 0.45	46.60 ± 2.07
	48	n.d.	9.83 ± 1.63	1.62 ± 0.32	6.10 ± 0.42	1.52 ± 0.46	48.89 ± 2.49
	60	n.d.	5.98 ± 1.47	1.72 ± 0.49	6.19 ± 0.45	1.59 ± 0.48	50.60 ± 2.22
	72	n.d.	3.47 ± 1.15	1.82 ± 0.41	6.15 ± 0.36	1.64 ± 0.47	52.08 ± 1.63
M6-6	Input	84.71 ± 0.98	36.01 ± 0.52	n.d.	n.d.	n.d.	n.d.
	0	82.97 ± 1.50	35.58 ± 0.84	n.d.	n.d.	n.d.	n.d.
	1	76.85 ± 1.13	35.12 ± 0.66	0.55 ± 0.56	1.22 ± 0.41	n.d.	3.52 ± 0.52
	3	54.44 ± 2.50	34.25 ± 0.60	0.61 ± 0.63	2.78 ± 0.40	0.98 ± 0.37	13.40 ± 0.82
	6	1.86 ± 1.11	32.19 ± 0.38	0.93 ± 0.46	4.96 ± 0.44	1.20 ± 0.38	37.88 ± 1.00
	12	n.d.	26.08 ± 0.01	1.01 ± 0.48	5.2 ± 0.47	1.30 ± 0.38	40.49 ± 1.50
	24	n.d.	20.26 ± 0.37	1.27 ± 0.34	5.93 ± 0.35	1.43 ± 0.34	45.05 ± 1.11

	36	n.d.	12.74 ± 0.08	1.53 ± 0.43	6.11 ± 0.35	1.51 ± 0.32	48.63 ± 0.85
	48	n.d.	7.25 ± 0.48	1.61 ± 0.35	6.23 ± 0.49	1.59 ± 0.37	51.00 ± 1.27
	60	n.d.	3.70 ± 0.78	1.74 ± 0.42	6.22 ± 0.50	1.65 ± 0.36	52.07 ± 0.84
	72	n.d.	1.96 ± 0.73	1.70 ± 0.53	6.14 ± 0.53	1.71 ± 0.37	52.56 ± 1.98
M6-7	Input	84.61 ± 1.14	35.86 ± 0.78	n.d.	n.d.	n.d.	n.d.
	0	82.55 ± 1.95	35.30 ± 1.24	n.d.	n.d.	n.d.	n.d.
	1	76.95 ± 1.76	34.88 ± 1.07	0.55 ± 0.56	1.16 ± 0.39	n.d.	3.32 ± 0.53
	3	56.75 ± 1.97	34.27 ± 1.18	0.61 ± 0.62	2.64 ± 0.37	0.94 ± 0.41	12.16 ± 0.38
	6	4.65 ± 1.20	32.19 ± 0.35	0.90 ± 0.36	4.83 ± 0.33	1.19 ± 0.42	36.37 ± 0.38
	12	n.d.	26.40 ± 1.05	0.96 ± 0.39	5.10 ± 0.38	1.23 ± 0.42	40.06 ± 1.07
	24	n.d.	20.77 ± 1.41	1.27 ± 0.33	5.99 ± 0.35	1.37 ± 0.39	44.89 ± 1.22
	36	n.d.	13.11 ± 2.10	1.55 ± 0.40	6.09 ± 0.38	1.42 ± 0.41	47.89 ± 1.66
	48	n.d.	7.44 ± 2.26	1.67 ± 0.35	6.25 ± 0.48	1.50 ± 0.43	50.08 ± 2.68
	60	n.d.	3.68 ± 1.44	1.77 ± 0.40	6.10 ± 0.42	1.54 ± 0.42	51.39 ± 1.16
	72	n.d.	1.91 ± 0.87	1.78 ± 0.26	6.16 ± 0.53	1.59 ± 0.43	52.13 ± 1.55
M6-10	Input	84.45 ± 1.39	35.89 ± 0.72	n.d.	n.d.	n.d.	n.d.
	0	83.44 ± 1.72	35.77 ± 0.86	n.d.	n.d.	n.d.	n.d.
	1	76.27 ± 0.43	34.82 ± 0.45	0.57 ± 0.57	1.23 ± 0.42	n.d.	3.55 ± 0.66
	3	55.26 ± 1.23	34.33 ± 0.51	0.64 ± 0.64	2.75 ± 0.55	0.69 ± 0.70	13.07 ± 1.33
	6	2.57 ± 0.50	31.74 ± 0.64	0.93 ± 0.40	4.94 ± 0.50	1.19 ± 0.32	37.66 ± 0.99
	12	n.d.	24.34 ± 1.23	1.11 ± 0.43	5.36 ± 0.56	1.3 ± 0.36	41.16 ± 1.08
	24	n.d.	13.74 ± 2.62	1.33 ± 0.43	6.1 ± 0.94	1.45 ± 0.36	47.14 ± 2.64
	36	n.d.	6.39 ± 4.01	1.55 ± 0.47	6.31 ± 0.80	1.50 ± 0.34	50.81 ± 3.02
	48	n.d.	2.99 ± 2.47	1.62 ± 0.43	6.34 ± 0.75	1.57 ± 0.35	52.15 ± 2.74
	60	n.d.	1.66 ± 1.24	1.66 ± 0.34	6.36 ± 0.43	1.63 ± 0.37	52.65 ± 1.38
	72	n.d.	0.83 ± 0.75	1.65 ± 0.33	6.29 ± 0.35	1.67 ± 0.37	52.62 ± 0.83
M6-11	Input	84.45 ± 1.39	35.89 ± 0.72	n.d.	n.d.	n.d.	n.d.
	0	82.61 ± 1.29	35.40 ± 0.81	n.d.	n.d.	n.d.	n.d.
	1	76.14 ± 1.14	34.77 ± 0.72	0.56 ± 0.57	1.23 ± 0.42	n.d.	3.65 ± 0.62
	3	53.53 ± 0.73	34.05 ± 0.51	0.63 ± 0.64	2.79 ± 0.49	0.98 ± 0.36	13.73 ± 1.08
	6	1.40 ± 0.65	32.3 ± 0.20	0.97 ± 0.44	5.08 ± 0.46	1.19 ± 0.34	39.00 ± 1.12

	12	n.d.	23.77 ± 0.88	1.09 ± 0.43	5.24 ± 0.56	1.30 ± 0.36	41.14 ± 1.53
	24	n.d.	14.93 ± 1.28	1.30 ± 0.55	5.93 ± 0.81	1.43 ± 0.36	46.54 ± 2.30
	36	n.d.	7.09 ± 1.88	1.55 ± 0.44	6.17 ± 0.73	1.52 ± 0.34	50.44 ± 2.81
	48	n.d.	3.09 ± 1.33	1.64 ± 0.40	6.20 ± 0.61	1.56 ± 0.33	51.85 ± 2.35
	60	n.d.	1.48 ± 0.59	1.73 ± 0.34	6.44 ± 0.41	1.65 ± 0.35	53.46 ± 1.56
	72	n.d.	0.86 ± 0.31	1.70 ± 0.35	6.25 ± 0.44	1.68 ± 0.36	52.96 ± 0.78
M6-13	Input	84.45 ± 1.39	35.89 ± 0.72	n.d.	n.d.	n.d.	n.d.
	0	83.69 ± 1.19	35.99 ± 0.72	n.d.	n.d.	n.d.	n.d.
	1	77.09 ± 0.64	35.24 ± 0.52	0.94 ± 0.33	1.48 ± 0.34	n.d.	3.87 ± 0.50
	3	56.64 ± 0.10	34.93 ± 0.99	1.06 ± 0.39	3.05 ± 0.49	1.23 ± 0.31	13.01 ± 1.05
	6	3.30 ± 0.51	32.43 ± 0.36	1.18 ± 0.30	5.25 ± 0.50	1.45 ± 0.29	37.78 ± 1.63
	12	n.d.	26.74 ± 0.05	1.32 ± 0.40	5.44 ± 0.43	1.52 ± 0.29	40.87 ± 1.31
	24	n.d.	19.85 ± 1.51	1.59 ± 0.45	6.29 ± 0.49	1.64 ± 0.31	45.79 ± 1.68
	36	n.d.	11.94 ± 3.12	1.83 ± 0.38	6.49 ± 0.46	1.71 ± 0.30	49.41 ± 2.04
	48	n.d.	6.55 ± 3.26	1.94 ± 0.34	6.53 ± 0.42	1.77 ± 0.30	50.87 ± 2.42
	60	n.d.	3.70 ± 2.25	2.00 ± 0.33	6.49 ± 0.47	1.82 ± 0.31	52.49 ± 2.04
	72	n.d.	2.38 ± 1.07	2.03 ± 0.32	6.50 ± 0.53	1.89 ± 0.34	53.61 ± 2.21
M6-15	Input	83.54 ± 1.05	35.44 ± 0.64	n.d.	n.d.	n.d.	n.d.
	0	82.50 ± 1.32	35.30 ± 0.85	n.d.	n.d.	n.d.	n.d.
	1	76.73 ± 1.00	34.99 ± 0.53	0.56 ± 0.55	1.22 ± 0.39	n.d.	3.56 ± 0.63
	3	54.6 ± 1.00	33.97 ± 0.42	0.63 ± 0.61	2.72 ± 0.39	0.97 ± 0.39	13.07 ± 0.87
	6	2.40 ± 1.34	31.87 ± 1.67	0.91 ± 0.35	4.92 ± 0.27	1.20 ± 0.38	37.73 ± 0.75
	12	n.d.	24.68 ± 0.67	1.10 ± 0.37	5.25 ± 0.32	1.29 ± 0.38	40.82 ± 0.98
	24	n.d.	15.58 ± 1.11	1.34 ± 0.42	5.99 ± 0.51	1.42 ± 0.38	46.21 ± 1.87
	36	n.d.	7.36 ± 1.14	1.57 ± 0.41	6.21 ± 0.56	1.51 ± 0.37	50.22 ± 2.63
	48	n.d.	3.11 ± 0.87	1.70 ± 0.36	6.42 ± 0.21	1.57 ± 0.37	52.78 ± 1.73
	60	n.d.	1.34 ± 0.32	1.69 ± 0.32	6.28 ± 0.21	1.62 ± 0.39	52.48 ± 1.24
	72	n.d.	0.84 ± 0.31	1.71 ± 0.37	6.27 ± 0.25	1.69 ± 0.41	53.09 ± 1.30
M6-16	Input	84.79 ± 1.63	35.97 ± 0.79	n.d.	n.d.	n.d.	n.d.
	0	83.29 ± 1.44	35.64 ± 0.71	n.d.	n.d.	n.d.	n.d.
	1	77.62 ± 1.16	35.29 ± 0.59	0.53 ± 0.49	1.18 ± 0.21	n.d.	3.33 ± 0.23

	3	56.13 ± 2.16	34.75 ± 0.75	0.78 ± 0.27	2.77 ± 0.06	0.96 ± 0.30	12.83 ± 0.22
	6	2.03 ± 1.97	33.18 ± 0.51	0.92 ± 0.22	5.17 ± 0.08	1.19 ± 0.32	37.71 ± 0.70
	12	n.d.	26.75 ± 0.96	1.10 ± 0.26	5.47 ± 0.2	1.28 ± 0.33	40.60 ± 1.22
	24	n.d.	17.72 ± 1.66	1.25 ± 0.19	5.73 ± 0.21	1.41 ± 0.30	44.24 ± 1.54
	36	n.d.	10.01 ± 2.43	1.41 ± 0.28	6.00 ± 0.29	1.51 ± 0.31	48.30 ± 2.75
	48	n.d.	5.65 ± 2.45	1.61 ± 0.14	6.29 ± 0.13	1.57 ± 0.32	50.65 ± 1.17
	60	n.d.	2.92 ± 1.37	1.67 ± 0.30	6.39 ± 0.18	1.60 ± 0.30	52.44 ± 1.78
	72	n.d.	1.51 ± 0.59	1.72 ± 0.32	6.34 ± 0.07	1.66 ± 0.32	52.98 ± 1.17
M6-19	Input	85.05 ± 1.19	36.09 ± 0.58	n.d.	n.d.	n.d.	n.d.
	0	84.67 ± 2.89	36.25 ± 1.35	n.d.	n.d.	n.d.	n.d.
	1	78.27 ± 0.68	35.60 ± 0.36	0.54 ± 0.51	1.19 ± 0.32	n.d.	3.33 ± 0.47
	3	55.72 ± 1.16	34.72 ± 0.69	0.61 ± 0.58	2.93 ± 0.36	0.98 ± 0.33	12.86 ± 0.76
	6	2.09 ± 0.69	32.81 ± 0.64	0.92 ± 0.31	5.33 ± 0.37	1.18 ± 0.28	37.65 ± 1.08
	12	0.28 ± 0.48	27.76 ± 0.35	1.03 ± 0.42	5.59 ± 0.48	1.26 ± 0.31	40.27 ± 1.43
	24	n.d.	20.80 ± 2.40	1.30 ± 0.37	5.87 ± 0.32	1.34 ± 0.33	43.14 ± 1.47
	36	n.d.	13.96 ± 3.59	1.40 ± 0.35	6.04 ± 0.30	1.42 ± 0.32	46.72 ± 1.56
	48	n.d.	8.89 ± 3.79	1.62 ± 0.44	6.04 ± 0.33	1.46 ± 0.33	48.68 ± 2.33
	60	n.d.	5.89 ± 3.58	1.75 ± 0.32	6.41 ± 0.36	1.53 ± 0.32	51.65 ± 3.47
	72	n.d.	3.62 ± 2.55	1.88 ± 0.33	6.36 ± 0.57	1.57 ± 0.33	51.95 ± 3.7
M6-20	Input	85.05 ± 1.19	36.09 ± 0.58	n.d.	n.d.	n.d.	n.d.
	0	83.55 ± 1.70	35.73 ± 0.83	n.d.	n.d.	n.d.	n.d.
	1	78.38 ± 1.72	35.52 ± 0.55	0.54 ± 0.51	1.17 ± 0.37	n.d.	3.18 ± 0.67
	3	57.32 ± 3.69	34.29 ± 1.10	0.61 ± 0.58	2.64 ± 0.60	0.94 ± 0.35	11.94 ± 2.09
	6	5.89 ± 4.20	32.73 ± 1.47	0.90 ± 0.37	5.05 ± 0.52	1.21 ± 0.29	36.28 ± 2.19
	12	n.d.	24.62 ± 1.30	1.08 ± 0.40	5.53 ± 0.68	1.33 ± 0.33	41.61 ± 1.31
	24	n.d.	13.99 ± 3.48	1.32 ± 0.40	6.18 ± 0.65	1.50 ± 0.35	46.93 ± 1.45
	36	n.d.	7.15 ± 3.69	1.51 ± 0.34	6.34 ± 0.50	1.60 ± 0.36	50.02 ± 1.72
	48	n.d.	3.38 ± 2.08	1.66 ± 0.32	6.57 ± 0.52	1.70 ± 0.34	52.40 ± 0.56
	60	n.d.	1.59 ± 0.89	1.68 ± 0.31	6.55 ± 0.37	1.76 ± 0.33	52.85 ± 1.27
	72	n.d.	0.67 ± 0.68	1.70 ± 0.33	6.53 ± 0.43	1.83 ± 0.34	53.35 ± 2.49
M6-21	Input	84.79 ± 1.63	35.97 ± 0.79	n.d.	n.d.	n.d.	n.d.

	0	83.37 ± 0.96	35.72 ± 0.53	n.d.	n.d.	n.d.	n.d.
	1	77.50 ± 0.43	35.28 ± 0.44	0.54 ± 0.50	1.18 ± 0.29	n.d.	3.34 ± 0.44
	3	55.71 ± 0.29	34.54 ± 0.59	0.62 ± 0.57	2.77 ± 0.39	0.95 ± 0.30	12.96 ± 0.78
	6	2.15 ± 1.06	32.35 ± 0.71	0.91 ± 0.31	5.06 ± 0.46	1.17 ± 0.29	37.63 ± 1.57
	12	n.d.	26.15 ± 0.51	1.10 ± 0.33	5.43 ± 0.51	1.28 ± 0.28	41.32 ± 1.32
	24	n.d.	16.95 ± 2.99	1.34 ± 0.33	5.88 ± 0.46	1.44 ± 0.27	45.52 ± 2.08
	36	n.d.	9.17 ± 3.87	1.52 ± 0.35	6.05 ± 0.60	1.52 ± 0.25	48.61 ± 3.23
	48	n.d.	5.12 ± 3.48	1.68 ± 0.26	6.39 ± 0.34	1.57 ± 0.25	51.22 ± 2.92
	60	n.d.	2.67 ± 1.91	1.72 ± 0.24	6.37 ± 0.33	1.63 ± 0.26	52.73 ± 1.97
	72	n.d.	1.46 ± 0.95	1.73 ± 0.21	6.40 ± 0.26	1.68 ± 0.26	53.11 ± 1.22
	Input	84.94 ± 1.37	35.94 ± 0.84	n.d.	n.d.	n.d.	n.d.
	0	83.02 ± 1.61	35.38 ± 0.89	n.d.	n.d.	n.d.	n.d.
	1	77.09 ± 2.69	34.84 ± 1.28	0.54 ± 0.50	1.13 ± 0.32	n.d.	3.18 ± 0.38
	3	57.02 ± 3.88	34.04 ± 1.21	0.60 ± 0.57	2.65 ± 0.44	0.93 ± 0.34	11.67 ± 1.33
	6	6.94 ± 5.74	32.66 ± 1.54	0.90 ± 0.33	5.02 ± 0.48	1.17 ± 0.30	35.03 ± 2.70
M6-22	12	n.d.	26.64 ± 2.13	1.09 ± 0.38	5.44 ± 0.47	1.24 ± 0.32	40.54 ± 1.53
	24	n.d.	18.74 ± 3.31	1.28 ± 0.47	5.90 ± 0.41	1.38 ± 0.31	44.64 ± 1.38
	36	n.d.	11.99 ± 4.79	1.47 ± 0.28	6.12 ± 0.43	1.45 ± 0.31	48.08 ± 2.73
	48	n.d.	7.84 ± 5.08	1.67 ± 0.37	6.25 ± 0.36	1.48 ± 0.34	49.79 ± 1.53
	60	n.d.	4.48 ± 3.51	1.84 ± 0.22	6.23 ± 0.31	1.54 ± 0.33	51.42 ± 0.72
	72	n.d.	2.59 ± 2.00	1.98 ± 0.33	6.23 ± 0.31	1.61 ± 0.33	52.24 ± 0.13
