# Supplementary material for: Molecular evolutionary engineering of xylose isomerase to improve its catalytic activity and performance of micro-aerobic glucose/xylose co-fermentation in Saccharomyces cerevisiae
Source: Biotechnol Biofuels. 2019 Jun 6;12:139. doi: 10.1186/s13068-019-1474-z (PMC6551904; doi:10.1186/s13068-019-1474-z)
Supplement: Supplementary file 7 — Additional file 7: Table S5. Metabolic profiles of recombinant S. cerevisiae strains expressing mutated LpXIs in glucose/xylose co-fermentation. [file 13068_2019_1474_MOESM7_ESM.pdf]

Strain	Time (h)	Glucose	Xylose	Xylitol	Glycerol	Acetate	Ethanol
SS81	Input	83.85 ± 3.25	35.62 ± 1.02	n.d.	n.d.	n.d.	n.d.
	0	83.19 ± 2.94	35.61 ± 0.74	n.d.	n.d.	n.d.	n.d.
	1	77.00 ± 2.90	35.21 ± 0.50	0.60 ± 0.53	1.48 ± 0.10	n.d.	4.12 ± 0.50
	3	54.51 ± 3.24	34.50 ± 0.46	1.02 ± 0.1	3.00 ± 0.21	0.43 ± 0.75	13.92 ± 1.47
	6	2.79 ± 0.79	31.73 ± 1.21	1.15 ± 0.11	5.08 ± 0.18	1.35 ± 0.11	37.37 ± 1.63
	12	0.30 ± 0.51	28.93 ± 1.01	1.30 ± 0.09	5.35 ± 0.14	1.37 ± 0.11	40.09 ± 1.33
	24	n.d.	24.07 ± 1.97	1.47 ± 0.08	5.36 ± 0.12	1.41 ± 0.09	41.13 ± 1.54
	36	n.d.	20.56 ± 2.37	1.69 ± 0.15	5.54 ± 0.22	1.42 ± 0.12	43.35 ± 1.82
	48	n.d.	16.94 ± 2.66	1.91 ± 0.16	5.68 ± 0.22	1.47 ± 0.13	45.05 ± 1.79
	60	n.d.	12.91 ± 2.75	2.07 ± 0.19	5.64 ± 0.36	1.49 ± 0.11	46.98 ± 1.94
	72	n.d.	9.52 ± 3.47	2.22 ± 0.20	5.77 ± 0.16	1.52 ± 0.11	49.20 ± 2.37
SS82	Input	83.85 ± 3.25	35.62 ± 1.02	n.d.	n.d.	n.d.	n.d.
	0	83.34 ± 3.30	35.65 ± 0.78	n.d.	n.d.	n.d.	n.d.
	1	76.34 ± 1.86	34.93 ± 0.03	0.89 ± 0.09	1.50 ± 0.09	n.d.	4.14 ± 0.53
	3	53.94 ± 3.45	34.34 ± 0.58	1.05 ± 0.08	3.10 ± 0.14	0.43 ± 0.75	14.09 ± 1.16
	6	2.81 ± 0.85	30.71 ± 0.99	1.20 ± 0.09	5.30 ± 0.10	1.34 ± 0.10	37.97 ± 1.21
	12	0.58 ± 0.51	24.28 ± 0.67	1.35 ± 0.11	5.49 ± 0.12	1.37 ± 0.10	41.46 ± 1.70
	24	n.d.	17.19 ± 2.02	1.58 ± 0.09	5.71 ± 0.07	1.41 ± 0.12	44.48 ± 0.95
	36	n.d.	11.15 ± 2.33	1.85 ± 0.12	6.00 ± 0.22	1.46 ± 0.11	47.68 ± 1.20
	48	n.d.	6.25 ± 1.63	1.91 ± 0.23	6.07 ± 0.33	1.48 ± 0.14	49.80 ± 1.38
	60	n.d.	3.14 ± 0.58	2.07 ± 0.17	6.18 ± 0.33	1.52 ± 0.11	51.67 ± 1.91
	72	n.d.	1.59 ± 0.52	2.12 ± 0.21	6.14 ± 0.21	1.56 ± 0.12	52.32 ± 0.57
SS89	Input	83.56 ± 2.13	35.77 ± 1.21	n.d.	n.d.	n.d.	n.d.
	0	81.94 ± 0.92	35.36 ± 0.27	n.d.	n.d.	n.d.	n.d.
	1	74.88 ± 0.67	35.07 ± 0.11	0.87 ± 0.07	1.50 ± 0.06	n.d.	4.29 ± 0.41
	3	52.52 ± 1.14	34.52 ± 0.29	0.99 ± 0.11	3.14 ± 0.15	n.d.	14.16 ± 0.33
	6	1.96 ± 0.19	30.59 ± 1.03	1.16 ± 0.11	5.15 ± 0.29	1.18 ± 0.04	37.47 ± 0.45
	12	0.34 ± 0.58	27.81 ± 1.04	1.29 ± 0.09	5.46 ± 0.20	1.21 ± 0.05	39.71 ± 0.63

	24	n.d.	23.20 ± 1.92	1.52 ± 0.11	5.62 ± 0.34	1.26 ± 0.07	42.41 ± 0.14
	36	n.d.	17.97 ± 2.04	1.74 ± 0.10	5.66 ± 0.22	1.29 ± 0.06	44.47 ± 1.35
	48	n.d.	12.94 ± 1.92	1.93 ± 0.15	5.66 ± 0.31	1.30 ± 0.05	46.33 ± 0.64
	60	n.d.	9.39 ± 2.11	2.04 ± 0.21	5.73 ± 0.28	1.33 ± 0.06	47.91 ± 0.75
	72	n.d.	6.01 ± 1.78	2.21 ± 0.19	5.93 ± 0.19	1.33 ± 0.04	50.20 ± 0.44
	Input	83.68 ± 3.39	35.38 ± 1.14	n.d.	n.d.	n.d.	n.d.
	0	82.68 ± 1.53	35.29 ± 0.27	n.d.	n.d.	n.d.	n.d.
	1	75.10 ± 2.19	34.64 ± 0.13	0.88 ± 0.11	1.56 ± 0.05	n.d.	4.48 ± 0.15
	3	52.26 ± 2.49	33.37 ± 0.69	0.97 ± 0.16	3.14 ± 0.20	0.43 ± 0.75	14.37 ± 0.32
	6	3.02 ± 0.75	30.69 ± 0.58	1.17 ± 0.17	5.47 ± 0.04	1.31 ± 0.13	38.12 ± 0.88
SS92	12	0.77 ± 0.05	23.51 ± 1.01	1.37 ± 0.18	5.68 ± 0.22	1.34 ± 0.12	42.37 ± 0.27
	24	n.d.	15.02 ± 1.37	1.60 ± 0.15	5.83 ± 0.13	1.38 ± 0.11	45.57 ± 1.03
	36	n.d.	8.04 ± 0.31	1.78 ± 0.24	6.11 ± 0.43	1.41 ± 0.13	48.82 ± 1.36
	48	n.d.	3.76 ± 0.37	1.91 ± 0.23	6.25 ± 0.39	1.44 ± 0.14	50.12 ± 1.52
	60	n.d.	1.67 ± 0.04	1.90 ± 0.24	5.98 ± 0.44	1.48 ± 0.14	50.94 ± 1.56
	72	n.d.	1.00 ± 0.08	1.99 ± 0.09	6.23 ± 0.30	1.53 ± 0.14	52.69 ± 0.40
	Input	82.68 ± 1.65	35.2 ± 0.84	n.d.	n.d.	n.d.	n.d.
	0	81.97 ± 1.06	35.3 ± 0.69	n.d.	n.d.	n.d.	n.d.
	1	75.63 ± 3.19	35.31 ± 0.97	0.87 ± 0.10	1.54 ± 0.02	n.d.	4.34 ± 0.30
	3	51.34 ± 3.08	33.93 ± 1.30	1.00 ± 0.10	3.19 ± 0.08	n.d.	14.47 ± 0.64
	6	1.37 ± 0.24	32.30 ± 0.21	1.21 ± 0.15	5.58 ± 0.15	1.30 ± 0.11	38.60 ± 0.66
SS93	12	0.82 ± 0.09	27.67 ± 0.41	1.30 ± 0.13	5.52 ± 0.10	1.32 ± 0.12	39.81 ± 0.41
	24	n.d.	22.85 ± 0.61	1.52 ± 0.13	5.67 ± 0.23	1.37 ± 0.12	42.25 ± 0.66
	36	n.d.	17.89 ± 1.05	1.72 ± 0.13	5.68 ± 0.16	1.38 ± 0.11	43.88 ± 0.75
	48	n.d.	13.52 ± 1.08	1.91 ± 0.15	5.75 ± 0.23	1.42 ± 0.14	45.78 ± 0.84
	60	n.d.	8.94 ± 1.46	2.02 ± 0.24	5.72 ± 0.39	1.45 ± 0.14	47.42 ± 0.37
	72	n.d.	5.85 ± 1.10	2.20 ± 0.24	5.92 ± 0.35	1.46 ± 0.16	49.24 ± 0.48
