# Supplementary material for: Molecular evolutionary engineering of xylose isomerase to improve its catalytic activity and performance of micro-aerobic glucose/xylose co-fermentation in Saccharomyces cerevisiae
Source: Biotechnol Biofuels. 2019 Jun 6;12:139. doi: 10.1186/s13068-019-1474-z (PMC6551904; doi:10.1186/s13068-019-1474-z)
Supplement: Supplementary file 9 — Additional file 9: Table S6. Metabolic profiles of recombinant S. cerevisiae strains expressed mutated LpXIs in a glucose/xylose co-fermentation. [file 13068_2019_1474_MOESM9_ESM.pdf]

Strain	Time (h)	Glucose	Xylose	Xylitol	Glycerol	Acetate	Ethanol
SS84	Input	87.59	36.70	n.d.	n.d.	n.d.	n.d.
	0	86.33	36.27	n.d.	n.d.	n.d.	n.d.
	1	77.96	35.03	1.02	1.63	n.d.	4.40
	3	54.21	34.46	1.17	3.35	n.d.	14.97
	6	2.14	31.93	1.39	5.51	1.40	38.78
	12	0.81	29.24	1.45	5.54	1.45	40.59
	24	n.d.	26.23	1.62	5.58	1.48	41.29
	36	n.d.	23.59	1.87	5.90	1.52	43.58
	48	n.d.	21.23	2.10	6.17	1.56	45.47
	60	n.d.	17.47	2.26	6.11	1.58	45.77
	72	n.d.	14.32	2.49	6.14	1.61	47.10
SS85	Input	84.59	36.18	n.d.	n.d.	n.d.	n.d.
	0	83.71	36.32	n.d.	n.d.	n.d.	n.d.
	1	78.13	35.75	0.96	1.53	n.d.	4.00
	3	54.36	35.56	1.15	3.14	1.32	14.23
	6	1.49	33.57	1.30	5.35	1.47	37.41
	12	0.89	32.67	1.35	5.46	1.52	38.28
	24	n.d.	31.13	1.48	5.54	1.58	38.26
	36	n.d.	27.78	1.60	5.38	1.57	40.12
	48	n.d.	25.98	1.75	5.50	1.60	40.46
	60	n.d.	23.85	1.92	5.61	1.65	42.53
	72	n.d.	21.68	2.06	5.68	1.69	43.32
SS86	Input	87.59	36.70	n.d.	n.d.	n.d.	n.d.
	0	85.76	36.07	n.d.	n.d.	n.d.	n.d.
	1	78.13	35.02	0.98	1.56	n.d.	4.24
	3	55.55	34.63	1.11	3.14	1.29	14.60
	6	3.92	31.88	1.24	5.21	1.44	38.26
	12	0.79	28.55	1.38	5.30	1.49	39.96

	24	n.d.	25.11	1.56	5.38	1.51	41.38
	36	n.d.	23.69	1.84	5.89	1.56	44.01
	48	n.d.	19.45	2.08	5.84	1.59	45.16
	60	n.d.	15.98	2.19	5.83	1.62	46.55
	72	n.d.	12.74	2.35	5.88	1.67	48.51
SS87	Input	81.71	34.99	n.d.	n.d.	n.d.	n.d.
	0	81.33	35.10	n.d.	n.d.	n.d.	n.d.
	1	75.06	34.87	0.81	1.44	n.d.	3.65
	3	51.59	34.83	0.88	3.06	1.24	15.51
	6	2.24	32.09	1.07	4.83	1.35	39.29
	12	n.d.	29.03	1.19	4.97	1.38	40.97
	24	n.d.	26.48	1.35	4.93	1.44	42.03
	36	n.d.	24.63	1.52	5.06	1.50	44.26
	48	n.d.	21.90	1.67	5.06	1.53	43.88
	60	n.d.	19.47	1.83	5.10	1.57	44.92
	72	n.d.	17.22	1.96	5.09	1.61	45.87
SS88	Input	87.59	36.70	n.d.	n.d.	n.d.	n.d.
	0	84.77	35.70	n.d.	n.d.	n.d.	n.d.
	1	78.18	35.32	1.06	1.58	n.d.	4.25
	3	55.24	34.55	1.15	3.28	1.29	14.59
	6	3.06	31.94	1.29	5.39	1.45	38.29
	12	0.80	28.60	1.41	5.42	1.47	40.11
	24	n.d.	24.46	1.58	5.40	1.50	42.10
	36	n.d.	23.55	1.88	5.98	1.54	43.51
	48	n.d.	19.81	2.09	6.05	1.56	44.57
	60	n.d.	16.33	2.24	6.03	1.59	45.95
	72	n.d.	12.79	2.42	6.12	1.62	47.96
SS91	Input	83.41	35.74	n.d.	n.d.	n.d.	n.d.
	0	83.78	36.16	n.d.	n.d.	n.d.	n.d.
	1	79.02	36.50	1.01	1.60	n.d.	4.05
	3	53.62	35.06	1.12	3.18	n.d.	1.35

	6	2.44	33.21	1.27	5.47	1.49	37.36
	12	0.97	30.81	1.34	5.47	1.53	38.81
	24	n.d.	26.75	1.50	5.49	1.59	39.37
	36	n.d.	23.41	1.70	5.59	1.59	41.00
	48	n.d.	21.10	1.96	6.05	1.69	44.54
	60	n.d.	16.59	2.15	6.00	1.68	45.84
	72	n.d.	13.34	2.32	6.02	1.73	47.30
SS94	Input	87.59	36.70	n.d.	n.d.	n.d.	n.d.
	0	84.67	35.53	n.d.	n.d.	n.d.	n.d.
	1	77.78	34.90	1.01	1.61	n.d.	4.33
	3	53.60	34.39	1.15	3.30	1.28	15.00
	6	3.85	32.83	1.29	5.29	1.43	38.35
	12	0.81	31.24	1.38	5.29	1.47	38.77
	24	n.d.	31.96	1.58	5.64	1.54	38.63
	36	n.d.	31.73	1.72	5.66	1.59	39.89
	48	n.d.	30.32	1.87	5.69	1.65	40.65
	60	n.d.	29.70	2.01	5.75	1.71	41.23
	72	n.d.	27.69	2.12	5.57	1.81	41.69
