# Supplementary material for: Molecular evolutionary engineering of xylose isomerase to improve its catalytic activity and performance of micro-aerobic glucose/xylose co-fermentation in Saccharomyces cerevisiae
Source: Biotechnol Biofuels. 2019 Jun 6;12:139. doi: 10.1186/s13068-019-1474-z (PMC6551904; doi:10.1186/s13068-019-1474-z)
Supplement: Supplementary file 10 — Additional file 10: Table S7. Metabolic profiles of recombinant S. cerevisiae strains expressed mutated LpXIs in a glucose/xylose co-fermentation. [file 13068_2019_1474_MOESM10_ESM.pdf]

Strain	Time (h)	Glucose	Xylose	Xylitol	Glycerol	Acetate	Ethanol
SS104	Input	85.13 ± 0.47	36.6 ± 0.37	n.d.	n.d.	n.d.	n.d.
	0	83.88 ± 1.10	36.64 ± 0.60	n.d.	n.d.	n.d.	0.59 ± 1.01
	1	77.47 ± 2.01	36.74 ± 0.79	1.01 ± 0.03	1.70 ± 0.07	n.d.	4.88 ± 0.60
	3	47.37 ± 3.22	34.76 ± 0.67	1.14 ± 0.06	3.74 ± 0.19	0.83 ± 0.72	17.20 ± 1.95
	6	1.72 ± 0.47	30.87 ± 1.32	1.29 ± 0.09	5.79 ± 0.30	1.35 ± 0.05	39.17 ± 1.72
	12	0.89 ± 0.05	26.01 ± 1.33	1.48 ± 0.07	5.89 ± 0.15	1.39 ± 0.05	41.09 ± 1.58
	24	n.d.	20.68 ± 1.18	1.72 ± 0.07	6.15 ± 0.26	1.44 ± 0.04	43.27 ± 1.84
	36	n.d.	15.38 ± 1.56	1.95 ± 0.06	6.28 ± 0.20	1.47 ± 0.04	45.57 ± 2.04
	48	n.d.	10.34 ± 1.70	2.17 ± 0.03	6.43 ± 0.15	1.50 ± 0.04	48.26 ± 1.85
	60	n.d.	6.08 ± 1.42	2.30 ± 0.08	6.51 ± 0.17	1.53 ± 0.05	50.10 ± 1.17
	72	n.d.	3.34 ± 0.84	2.41 ± 0.06	6.57 ± 0.10	1.59 ± 0.04	52.29 ± 1.24
SS105	Input	81.17 ± 0.00	34.67 ± 0.00	n.d.	n.d.	n.d.	n.d.
	0	81.04 ± 0.49	34.90 ± 0.19	n.d.	n.d.	n.d.	n.d.
	1	75.32 ± 0.28	34.87 ± 0.20	0.84 ± 0.01	1.51 ± 0.01	n.d.	3.65 ± 0.11
	3	49.02 ± 0.95	34.28 ± 0.25	0.93 ± 0.01	3.36 ± 0.07	n.d.	16.36 ± 0.24
	6	2.27 ± 0.50	31.84 ± 0.47	1.10 ± 0.03	5.17 ± 0.04	1.3 ± 0.01	39.07 ± 0.53
	12	n.d.	29.62 ± 0.88	1.24 ± 0.03	5.21 ± 0.06	1.34 ± 0.01	41.10 ± 0.53
	24	n.d.	26.29 ± 1.72	1.45 ± 0.07	5.38 ± 0.14	1.4 ± 0.02	41.82 ± 0.71
	36	n.d.	23.33 ± 2.23	1.61 ± 0.06	5.34 ± 0.15	1.44 ± 0.03	43.40 ± 0.85
	48	n.d.	21.20 ± 1.30	1.75 ± 0.12	5.4 ± 0.10	1.49 ± 0.00	44.27 ± 1.36
	60	n.d.	18.33 ± 1.82	1.89 ± 0.10	5.34 ± 0.03	1.51 ± 0.01	45.01 ± 0.57
	72	n.d.	16.50 ± 1.71	2.03 ± 0.11	5.43 ± 0.09	1.57 ± 0.02	46.10 ± 0.61
SS120	Input	85.40 ± 0.00	36.81 ± 0.00	n.d.	n.d.	n.d.	n.d.
	0	84.38 ± 1.91	36.93 ± 0.73	n.d.	n.d.	n.d.	1.19 ± 1.03
	1	77.02 ± 1.86	36.88 ± 0.65	0.97 ± 0.01	1.69 ± 0.05	n.d.	5.14 ± 0.16
	3	46.96 ± 2.83	35.08 ± 0.61	1.08 ± 0.06	3.63 ± 0.12	1.25 ± 0.01	18.27 ± 0.58
	6	1.52 ± 0.43	30.04 ± 1.16	1.29 ± 0.02	5.65 ± 0.06	1.34 ± 0.01	40.75 ± 0.53
	12	0.91 ± 0.04	23.73 ± 1.12	1.43 ± 0.03	5.82 ± 0.16	1.38 ± 0.01	43.04 ± 0.69

24	n.d.	16.08 ± 1.69	1.72 ± 0.03	6.19 ± 0.16	1.43 ± 0.02	45.82 ± 1.21
36	n.d.	9.69 ± 1.94	1.93 ± 0.02	6.30 ± 0.16	1.45 ± 0.02	48.64 ± 0.60
48	n.d.	5.00 ± 1.55	2.06 ± 0.01	6.40 ± 0.13	1.49 ± 0.01	51.02 ± 0.65
60	n.d.	2.33 ± 0.79	2.06 ± 0.11	6.43 ± 0.10	1.54 ± 0.01	52.29 ± 0.69
72	n.d.	1.34 ± 0.30	2.17 ± 0.04	6.51 ± 0.18	1.61 ± 0.02	53.27 ± 0.92
