# Supplementary material for: Molecular evolutionary engineering of xylose isomerase to improve its catalytic activity and performance of micro-aerobic glucose/xylose co-fermentation in Saccharomyces cerevisiae
Source: Biotechnol Biofuels. 2019 Jun 6;12:139. doi: 10.1186/s13068-019-1474-z (PMC6551904; doi:10.1186/s13068-019-1474-z)

Strain	LpXI	n	Concentrations of xylose 500 mM	100 mM	50 mM	10 mM
SS81	Wild-type	1	0.0133	0.0079	0.0065	0.0024
		2	0.0134	0.0080	0.0066	0.0024
		3	0.0132	0.0071	0.0061	0.0022
		mean	0.0133	0.0077	0.0064	0.0023
		sd	0.0001	0.0005	0.0003	0.0001
SS82	T63I/A121A	1	0.0145	0.0156	0.0110	0.0047
		2	0.0145	0.0152	0.0109	0.0043
		3	0.0148	0.0150	0.0103	0.0042
		mean	0.0146	0.0153	0.0107	0.0044
		sd	0.0002	0.0003	0.0004	0.0003
SS92	V162A/T273T/N303T	1	0.0104	0.0120	0.0074	0.0034
		2	0.0105	0.0110	0.0080	0.0032
		3	0.0105	0.0107	0.0078	0.0031
		mean	0.0105	0.0112	0.0077	0.0032
		sd	0.0001	0.0007	0.0003	0.0002
SS104	V162A	1	0.0098	0.0113	0.0068	0.0031
		2	0.0100	0.0104	0.0074	0.0029
		3	0.0103	0.0103	0.0074	0.0030
		mean	0.0100	0.0107	0.0072	0.0030
		sd	0.0003	0.0006	0.0003	0.0001
SS105	N303T	1	0.0048	0.0062	0.0040	0.0020
		2	0.0048	0.0051	0.0039	0.0019
		3	0.0050	0.0054	0.0039	0.0019
		mean±SD	0.0049	0.0056	0.0039	0.0019
		sd	0.0001	0.0006	0.0001	0.0001
SS120	T63I/V162A	1	0.0161	0.0165	0.0118	0.0041
		2	0.0161	0.0162	0.0116	0.0043
		3	0.0166	0.0158	0.0118	0.0043
		mean±SD	0.0163	0.0162	0.0117	0.0042
		sd	0.0003	0.0004	0.0001	0.0001
		1/[S]	0.0020	0.0100	0.0200	0.1000

Strain	LpXI	n	1/n 500 mM	100 mM	50 mM	10 mM
SS81	Wild-type	1	75.18796992	126.58227848	153.84615385	416.66666667
		2	74.62686567	125.00000000	151.51515152	416.66666667
		3	75.75757576	140.84507042	163.93442623	454.54545455
		mean	75.18796992	130.43478261	156.25000000	428.57142857
		sd	0.56536037	8.72732391	6.60107562	21.86932838
SS82	T63I/A121A	1	68.96551724	64.10256410	90.90909091	212.76595745
		2	68.96551724	65.78947368	91.74311927	232.55813953
		3	67.56756757	66.66666667	97.08737864	238.09523810
		mean	68.49315068	65.50218341	93.16770186	227.27272727
		sd	0.80710662	1.30318542	3.35231141	13.31642088
SS92	V162A/T273T/N303T	1	96.15384615	83.33333333	135.13513514	294.11764706
		2	95.23809524	90.90909091	125.00000000	312.50000000
		3	95.23809524	93.45794393	128.20512821	322.58064516
		mean	95.54140127	89.02077151	129.31034483	309.27835052
		sd	0.52870904	5.26618923	5.18039263	14.43186617
SS104	V162A	1	102.04081633	88.49557522	147.05882353	322.58064516
		2	100.00000000	96.15384615	135.13513514	344.82758621
		3	97.08737864	97.08737864	135.13513514	333.33333333
		mean	99.66777409	93.75000000	138.88888889	333.33333333
		sd	2.48947248	4.71415745	6.88414470	11.12553023
SS105	N303T	1	208.33333333	161.29032258	250.00000000	500.00000000
		2	208.33333333	196.07843137	256.41025641	526.31578947
		3	200.00000000	185.18518519	256.41025641	526.31578947
		mean±SD	205.47945205	179.64071856	254.23728814	517.24137931
		sd	4.81125224	17.79438013	3.70096326	15.19342814
SS120	T63I/V162A	1	62.11180124	60.60606061	84.74576271	243.90243902
		2	62.11180124	61.72839506	86.20689655	232.55813953
		3	60.24096386	63.29113924	84.74576271	232.55813953
		mean±SD	61.47540984	61.85567010	85.22727273	236.22047244
		sd	1.08012847	1.34854559	0.84358602	6.54963436

Strain	LpXI	n	Km/Vmax	1/Vmax (340 nm)	Vmax (340 nm)	**Vmax (umol/min/mg protein)**	**Km**
SS81	Wild-type	1	3362.792322	82.09862061	0.0122	0.0650	41.0
		2	3376.469886	80.52866471	0.0124	0.0662	41.9
		3	3698.028049	86.73570612	0.0115	0.0615	42.6
		mean	3479.0968	83.1210	0.0120	**0.064**	**41.8**
		sd	189.7234	3.2273	0.0005	**0.002**	**0.8**
SS82	T63I/A121A	1	1538.908744	58.40179387	0.0171	0.0913	26.4
		2	1746.526654	57.12868285	0.0175	0.0934	30.6
		3	1799.335898	57.9761281	0.0172	0.0920	31.0
		mean	1694.9238	57.8355	0.0173	**0.092**	**29.3**
		sd	137.6689	0.6481	0.0002	**0.001**	**2.6**
SS92	V162A/T273T/N303T	1	2122.916347	82.12875098	0.0122	0.0649	25.8
		2	2320.951522	79.32039631	0.0126	0.0672	29.3
		3	2414.496242	80.19207714	0.0125	0.0665	30.1
		mean	2286.1214	80.5471	0.0124	**0.066**	**28.4**
		sd	148.8777	1.4374	0.0002	**0.001**	**2.3**
SS104	V162A	1	2358.893133	87.20049166	0.0115	0.0612	27.1
		2	2608.190152	82.95886684	0.0121	0.0643	31.4
		3	2494.121361	83.35480151	0.0120	0.0640	29.9
		mean	2487.0682	84.5047	0.0118	**0.063**	**29.5**
		sd	124.7981	2.3430	0.0003	**0.002**	**2.2**
SS105	N303T	1	3266.426358	172.1138442	0.0058	0.0310	19.0
		2	3409.513225	184.2705162	0.0054	0.0289	18.5
		3	3492.284532	176.7324182	0.0057	0.0302	19.8
		mean±SD	3389.4080	177.7056	0.0056	**0.030**	**19.1**
		sd	114.2635	6.1365	0.0002	**0.001**	**0.6**
SS120	T63I/V162A	1	1938.900987	48.85778333	0.0205	0.1092	39.7
		2	1807.984185	50.98783	0.0196	0.1046	35.5
		3	1814.66076	50.32519626	0.0199	0.1060	36.1
		mean±SD	1853.8486	50.0569	0.0200	**0.107**	**37.1**
		sd	73.7331	1.0901	0.0004	**0.002**	**2.3**

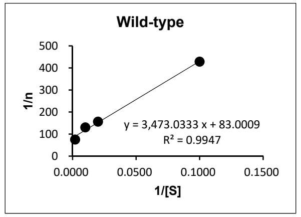

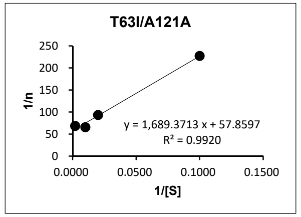

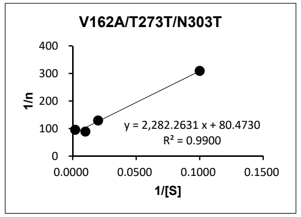

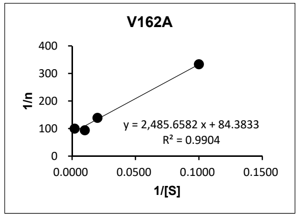

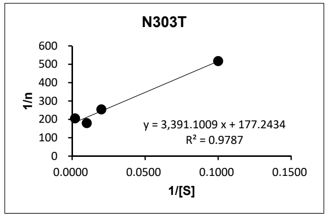

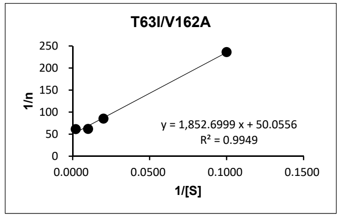

Supplement: Supplementary file 11 — Additional file 11: Table S8. Kinetic analysis of mutated LpXIs [file 13068_2019_1474_MOESM11_ESM.pdf]
