# Supplementary material for: Molecular evolutionary engineering of xylose isomerase to improve its catalytic activity and performance of micro-aerobic glucose/xylose co-fermentation in Saccharomyces cerevisiae
Source: Biotechnol Biofuels. 2019 Jun 6;12:139. doi: 10.1186/s13068-019-1474-z (PMC6551904; doi:10.1186/s13068-019-1474-z)
Supplement: Supplementary file 12 — Additional file 12: Table S9. Oligonucleotides used in this study. [file 13068_2019_1474_MOESM12_ESM.pdf]

Oligonucleotide	Sequence (5' > 3')
Kan exu F NdeI	GCGCGC*CATATG* TAGGTCTAGAGATCTGTTTAGCTTGCC
Kan exu R KpnI	GCGC*GGTACC* ATTAAGGGTTCTCGAGAGCTCGT
PspXIopt_F1	ATGGCTAAGGAATACTTCCCAC
PspXIopt_R1 XhoI	GCGC*CTCGAG* TTGGTACATAGCGACGATAGCTT
OspXIopt_F1	ATGACTAAGGAATACTTCCCAACTAT
OspXIopt_R1 XhoI	GCGC*CTCGAG* TTGGTACATAGCGACGATAGCTT
LpXIopt_F1	ATGAAGAACTACTTCCCAAACGTCC
LpXIopt_R1 XhoI	GCGC*CTCGAG* TCTGAACAAGATGTTGTTGACGA
RfXIopt_F1	ATGGAATTCTTCTCTAACATCGG
RfXIopt_R1 XhoI	GCGC*CTCGAG* CAAAGAGAACAAGACGTTGTTGAC
PrXIopt_F1	ATGGCTAAGGAATACTTCCCA
PrXIopt_R1 XhoI	GC*CTCGAG* CTTACAGTACAAAGCGACAGTAGTTTC
BcXIopt_F1	ATGTCTTACTTCGAACACATCCC
BcXIopt_R1 XhoI	GCGC*CTCGAG* ACGCAAACCGTAGATAGCTTG
RcXIopt_F1	ATGTCTGAAGTCTTCTCTGGTATCTC
RcXIopt_R1 XhoI	GCGC*CTCGAG* CTTAGTTTCCAAGATGTATTGGTTC
SrXIopt_F1	ATGAACTACCAACCAACTCCAGA
SrXIopt_R1 XhoI	GCGC*CTCGAG* ACCTCTAGCACCCAACAAGTG
TCYC1-PPGK1 F	TTTGCGGCCGGTACCACTAGTACTGTAATTGCTTT
pUG35-TCYC1R	TCGAGAACCCTTAATGGTACCGGCCGCAAATTAAA
oSS62 XI-F_HSP12s	ACTCAAAACAAAAAAAACTAAATACAACACCCAT
oSS74 XI-Rc	TTGGGAATTGGTCAGTGTCCCAAC
oSS63 XI-R_HSP12as	AGTTTTTTTTGTTTTGAGTTGTTTGTTTGAGATT
oSS83 06_LpXI-Fc	CTGACCAATTCCCAACTGACGTC
LpXIopt_Seq_F1	TTGGCTTTCTTGAGAAAG
LpXIopt_Seq_R1	TAGCGTGGTTAGCTTCGA
oSS106 06_LpXI_V162A	TGTAACGCTGACGCTTTCGCTTACGCT
oSS107 06_LpXI_V162Aas	AGAAGTAGAAGCACCGTGCATGAATCTTGG
oSS108 06_LpXI_N303T	CAAGGTGACCCAACTTTGGGTTGGGAC
oSS109 06_LpXI_N303Tas	GTTAGCGTCGACAGAACCGAAAGCACC

Restriction endonuclease sites are shown italicized and underlined.
